# Regulation of MRGPRX2-mediated mast cell function by competence-stimulating peptide 1 and pro-adrenomedullin peptide

**DOI:** 10.3389/fimmu.2026.1781889

**Published:** 2026-03-17

**Authors:** Aetas Amponnawarat, Sangita Sutradhar, Chalatip Chompunud Na Ayudhya, Maram Bawazir, Ronit Sagi-Eisenberg, Hydar Ali

**Affiliations:** 1 Department of Basic and Translational Sciences, University of Pennsylvania, School of Dental Medicine, Philadelphia, PA, United States; 2 Department of Advanced General Dentistry and Dental Public Health, Faculty of Dentistry, Chiang Mai University, Chiang Mai, Thailand; 3 Department of Oral Diagnosis, Faculty of Dentistry, Naresuan University, Phitsanulok, Thailand; 4 Department of Oral Diagnostic Sciences, Faculty of Dentistry, King Abdulaziz University, Jeddah, Saudi Arabia; 5 Department of Cell and Developmental Biology, Gray Faculty of Medical and Health Sciences, Tel Aviv University, Tel Aviv, Israel

**Keywords:** balanced agonist, biased agonist, CSP-1, mast cells, MRGPRX2, MRGPRX2 phosphorylation, PAMP-12

## Abstract

**Introduction:**

Mas-related G protein-coupled receptor (GPCR)-X2 (MRGPRX2, mouse ortholog MrgprB2) is predominantly expressed in mast cells (MCs) and is activated by a wide range of cationic ligands such as bacterial quorum sensing molecules (QSMs) and pro-adrenomedullin peptide 9-20 (PAMP-12). Activation of MrgprB2 by competence-stimulating peptide-1 (CSP-1), a QSM produced by Gram-positive bacteria, has recently been shown to promote antibacterial immunity, whereas its activation by PAMP-12 has been implicated in allergic contact dermatitis (ACD) and itch. Mechanisms via which the activation of the same receptor by different agonists contributes to different functional outcomes are unknown. GPCR agonists that activate both the G protein and receptor phosphorylation-mediated β-arrestin pathways are known as balanced agonists but agonists that activate only the G proteins are known as G protein-biased agonists. The goals of this study were to determine if CSP-1 and PAMP-12 serve as balanced or biased MRGPRX2 agonists and to investigate the differences in receptor internalization and desensitization in response to these agonists.

**Methods:**

Bioluminescence resonance energy transfer 2 (BRET2) in HEK293T cells expressing MRGPRX2 was utilized to investigate G protein coupling following MRGPRX2 activation. β-arrestin recruitment studies were performed using Transcriptional Activation Following Arrestin Translocation (Tango) assay. Effects of PAMP-12 and CSP-1 on MRGPRX2 phosphorylation, desensitization, receptor internalization, extracellular-signal-regulated kinase (ERK) phosphorylation and degranulation were determined in RBL cells stably expressing MRGPRX2.

**Results:**

PAMP-12 and CSP-1 coupled to Gαq, Gαi1, and Gαi3 and induced ERK phosphorylation and degranulation at similar levels. However, PAMP-12 caused MRGPRX2 phosphorylation and desensitization, but CSP-1 did not. PAMP-12 caused ~50-fold increase in β-arrestin recruitment and this was associated with ~60% internalization of cell surface MRGPRX2. By contrast, CSP-1 caused ~5-fold increase in β-arrestin recruitment and ~20% receptor internalization.

**Conclusion:**

These findings demonstrate that PAMP-12 and CSP-1 utilize shared G proteins to induce ERK phosphorylation and degranulation. However, they display substantial differences in their ability to cause MRGPRX2 phosphorylation, β-arrestin recruitment, receptor internalization and desensitization. These differences in G protein-biased and balanced signaling may dictate the ability of CSP-1 and PAMP-12 to contribute to host defense and ACD, respectively.

## Introduction

Mast cells (MCs) are well-recognized for their role in IgE/FcεRI-mediated allergic responses. However, they also contribute to host defense and non-IgE-mediated inflammatory disorders (1). All MCs are characterized by the expression of cell surface FcεRI, but human skin MCs express a novel G protein-coupled receptor (GPCR) known as Mas-related GPCR-X2 (MRGPRX2) at high levels (2, 3). However, MRGPRX2 is expressed at low levels in lung and gut MCs and the possibility that it is expressed in basophils and eosinophils has been the subject of controversy (2, 4, 5). Mouse skin and peritoneal MCs express MrgprB2, which has been designated as the ortholog of human MRGPRX2 (6). Despite the fact that they share only ~53% sequence homology, both receptors are activated by the same group of cationic agonists such as the polymer compound 48/80 (C48/80), neuropeptides, bacterial quorum sensing molecules (QSMs), host defense antimicrobial peptides (HDPs), pro-adrenomedullin peptide 9-20 (PAMP-12), and wide range of U.S. Food and Drug Administration (FDA)-approved drugs (3, 6). Studies with human MCs expressing MRGPRX2 as well as *Mrgprb2*^−/−^ mice have strongly implicated the role of these receptors in host defense against microbial infection. Competence-stimulating peptide 1 (CSP-1), a QSM produced by Gram-positive bacteria, has recently been shown to activate MCs via MRGPRX2/B2 to release antibacterial mediators and inhibit bacterial growth and biofilm formation *in vitro* (7). Moreover, studies using wild-type and *Mrgprb2^−/−^* mice showed that an enhanced bacterial clearance and improved disease score following exogenous CSP-1 treatment is MrgprB2-dependent, suggesting that MrgprB2-mediated MC activation by CSP-1 facilitates host protection against bacterial infection (7). Besides host defense, MrgprB2 contributes to allergic contact dermatitis (ACD) and non-histaminergic itch in mice (8, 9). These conditions are likely mediated via the activation of MRGPRX2 and MrgprB2 by PAMP-12, an endogenous peptide produced by keratinocytes. However, the molecular mechanisms via which activation of the same receptor by different agonists contribute to host protection versus disease remain unknown.

In addition to G proteins, most GPCR agonists activate an additional signaling pathway that involves receptor phosphorylation, leading to the recruitment of adaptor proteins known as β-arrestins (10). This pathway is well-characterized for its role in GPCR desensitization (uncoupling of the G-protein from the cognate receptor) and internalization (11). More recently, it has been shown that the β-arrestin pathway also performs an important role in G protein-independent downstream signaling for cell migration, growth, and differentiation (12, 13). GPCR agonists that preferentially activate either G proteins or β-arrestins are known as G protein-biased and β-arrestin-biased agonists, respectively. However, agonists that activate both pathways are known as balanced agonists (14, 15). Whether CSP-1 and PAMP-12 serve as balanced or biased agonists for MRGPRX2 has yet to be determined.

Biased agonism has gained tremendous attention due to its potential therapeutic implications for many GPCRs (16–19). For example, G protein-biased β_2_-adrenergic receptor (β_2_AR) agonists have been widely utilized for the treatment of asthma and obstructive lung diseases owing to their enhanced bronchodilation in the airway smooth muscle, in comparison to the balanced agonists (18, 19). Moreover, β-arrestin-biased β_1_-adrenergic receptor (β_1_AR) agonists improve cardiopathy treatment outcomes when compared to balanced agonists (18). These examples of biased agonism in βARs suggest that biased signaling in MRGPRX2 may also lead to the development of therapeutic strategies for MRGPRX2-mediated disorders. Although several balanced and G protein-biased agonists for MRGPRX2 have been identified (20–23), the significance of this difference is not clear. The goals of this study were to determine if CSP-1 and PAMP-12 serve as balanced or biased MRGPRX2 agonists and to investigate the differences in receptor phosphorylation, internalization, and desensitization in response to these agonists. The data presented herein demonstrate that both PAMP-12 and CSP-1 activate the same set of G proteins to induce similar levels of extracellular-signal-regulated kinase (ERK) phosphorylation and degranulation but display distinct differences in their ability to cause MRGPRX2 phosphorylation, β-arrestin recruitment, MRGPRX2 internalization, and desensitization.

## Materials and methods

### Materials

All cell culture reagents were obtained from Invitrogen (Gaithersburg, MD, USA). Competence-stimulating peptide 1 (CSP-1) was purchased from AnaSpec (Catalog #AS-63779). PAMP-12 (Catalog #HY-P2198) was obtained from MedChem Express. Compound 48/80 (C48/80; Catalog #C2313), 4-Nitrophenyl N-acetyl-β-D-glucosaminide (PNAG; Catalog #N9376), and dimethyl sulfoxide (DMSO) were purchased from Sigma-Aldrich (St. Louis, MO, USA). 2,4-Dinitrophenylated Bovine serum albumin (DNP-BSA; Catalog #A23018) was purchased from Invitrogen. Pertussis toxin (PTx) was obtained from List Biological Laboratories (Catalog #181). TRUPATH and MRGPRX2-Tango plasmid were gifts from Bryan Roth (Addgene kit #1000000163) (Addgene plasmid #66440; http://n2t.net/addgene:66440; RRID: Addgene_66440). Lipofectamine™ 2000 transfection reagent was obtained from Invitrogen. Fura-2 acetoxymethyl ester (Catalog #ab120873) and Mounting Medium with DAPI (Fluoroshield; Catalog #ab104139) were purchased from Abcam. Bright-Glo Luciferase was purchased from Promega (Catalog #E2620). Dyngo-4a and Cycloheximide were obtained from Cayman Chemical (Catalog #29479 and #14126). Phycoerythrin (PE)-conjugated mouse anti-MRGPRX2 antibody (Clone K125H4; Catalog #259004), unconjugated mouse anti-MRGPRX2 antibody (Clone K125H4; Catalog #359002), unconjugated rabbit anti-HA antibody (Clone Poly9023; Catalog #923501), and Intracellular Staining Perm Wash Buffer (Catalog #421002) were purchased from BioLegend. AF647-conjugated donkey anti-mouse IgG (Catalog #A31571), AF488-conjugated donkey anti-mouse IgG (Catalog #A21202), and AF488-conjugated goat anti-rabbit IgG (Catalog #11008) were purchased from Invitrogen. The unconjugated mouse anti-HA antibody was obtained from Roche (Clone 12CA5; Catalog #11583816001). The unconjugated rabbit anti-Phospho-p44/42 MAPK (pERK1/2) (Clone D13.14.4E; Catalog #4370S), anti-p44/42 MAPK (ERK1/2) (Catalog #4695S), anti-β-Actin (Clone 13E5, Catalog #4950S) monoclonal antibodies, HRP-linked anti-rabbit IgG (Catalog #7074S) and HRP-linked anti-mouse IgG (Catalog #7076S), and protein A agarose (50% bead slurry; Catalog #9863S) were purchased from Cell Signaling Technology. Unconjugated rabbit anti-Phospho-MRGPRX2 (Ser313, Ser327/328) polyclonal antibody was obtained from 7TM Antibodies (Catalog #7tm0157B).

### Cell culture

Rat basophilic leukemia (RBL-2H3) and HEK-293T cells were maintained as monolayer cultures in Dulbecco’s modified Eagle’s medium (DMEM) supplemented with GlutaMAX™, 10% fetal bovine serum (FBS), penicillin (100 IU/mL) and streptomycin (100 μg/mL) at 37 °C with 5% CO_2_. RBL-2H3 cells stably expressing MRGPRX2 were maintained similarly in the presence of 1 mg/mL G418. HTLA-MRGPRX2 cells (HEK-293T cells stably expressing a tTA-dependent luciferase reporter and a β-arrestin2-TEV fusion gene and MRGPRX2) were maintained in DMEM supplemented with GlutaMAX™, 10% FBS, penicillin (100 IU/mL), streptomycin (100 μg/mL), hygromycin (200 μg/mL), puromycin (5 μg/mL), and G418 (500 μg/mL) at 37 °C with 5% CO_2_ (24). Laboratory of Allergic Diseases 2 (LAD2) cells were supplied by Dr. A. Kirshenbaum and Dr. D. Metcalfe (National Institute of Allergy and Infectious Diseases, National Institutes of Health, Bethesda, MD, USA). Cells were cultured in a complete StemPro-34 medium supplemented with L-glutamine (2 mM), penicillin (100 IU/mL), streptomycin (100 μg/mL), and 100 ng/mL recombinant human stem cell factor (rhSCF). Cells culture was maintained by weekly hemidepletions using complete media containing rhSCF as described previously (25).

### Agonist-induced G protein activation: bioluminescence resonance energy transfer 2 assay

BRET2 assay was used to determine the effects of agonists on the activation of different G proteins, as described previously, but with slight modifications (26). Upon agonist-induced activation of MRGPRX2, heterotrimeric G proteins undergo conformational rearrangement and/or dissociation, leading to changes in the BRET2 signal that reflect G protein activation. Briefly, HEK293T cells were plated in 6-well plates at a density of 0.8-1×10^6^ cells/well and cultured overnight. The next day, cells were transfected in OptiMEM medium with a 1:1:1:1 DNA ratio of MRGPRX2:Gα-Rluc8:Gβ:Gγ-GFP2 plasmids (TRUPATH biosensors) with a total concentration of 2 µg DNA using Lipofectamine™ 2000 and cells were plated (50,000 cells/well) in a poly-L-lysine coated 96-well clear-bottomed, white-walled microplates. The following day, white backing stickers (Perkin Elmer, Waltham, MA) were applied to the bottom surface of the entire plate to prevent light scattering, and the medium was carefully aspirated and replaced immediately with 60 μL of assay buffer (1x HBSS + 20 mM HEPES, pH 7.4), followed by a 10 μL addition of freshly prepared 50 μM coelenterazine 400a (Gold Biotechnology Inc.). After 5 min, cells were stimulated with 30 μL of C48/80, CSP-1, or PAMP-12 at the indicated concentrations, and the plate was analyzed using a Varioskan™ LUX Multimode Microplate Reader (Thermo Scientific) with 395 nm (Rluc8-coelenterazine 400a) and 510 nm (GFP2) emission filters. BRET2 ratios were calculated as the ratio of the GFP2 to Rluc8 emission. Net BRET2 was determined by subtracting the BRET2 ratio of vehicle control from that of agonist-treated cells.

### Calcium mobilization assay

RBL-MRGPRX2 cells (2×10^6^) were loaded with 1 μM Fura-2 acetoxymethyl ester for 30 min at 37 °C, followed by de-esterification period in HEPES-containing buffer with 0.1% BSA for an additional 15 min at room temperature. Cells were washed, resuspended in 1.5 mL of the buffer, and then stimulated with C48/80, CSP-1, or PAMP-12. Calcium mobilization was determined using a Hitachi F-2700 Fluorescence Spectrophotometer with dual excitation wavelengths of 340 and 380 nm, and an emission wavelength of 510 nm.

For desensitization experiments, RBL-MRGPRX2 cells (2×10^6^) were preincubated with the indicated concentrations of C48/80, CSP-1, or PAMP-12 for the specified durations, prior to assessment of calcium mobilization using the protocol described above.

### Degranulation measured by β-hexosaminidase release assay

RBL-2H3 and RBL-MRGPRX2 (5 × 10^4^ cells/well) were seeded in a 96-well cell culture plate and cultured overnight in a 37 °C incubator with 5% CO_2_. Cells were washed twice and then suspended in 45 μL of fresh HEPES-containing buffer with 0.1% of bovine serum albumin (BSA). Experimental groups were stimulated with C48/80, CSP-1, PAMP-12, or DNP-BSA for 30 min at 37 °C. Cells treated with vehicle were designated as controls. To determine the total β-hexosaminidase release, unstimulated cells were lysed in 50 μL of 0.1% Triton X-100. Aliquots (20 μL) of supernatants or cell lysates were incubated with 20 μL of 1 mM PNAG for 1 h at 37 °C. The reaction was stopped by adding 250 μL of carbonate-bicarbonate stop buffer (0.1 M Na_2_CO_3_/0.1 M NaHCO_3_), prepared by dissolving 1.06 g Na_2_CO_3_ and 0.84 g NaHCO_3_ in distilled water to a final volume of 100 mL (pH ~10.0). β-hexosaminidase release was assessed by measuring the absorbance at 405 nm using a Versamax microplate spectrophotometer (Molecular Devices, San Jose, CA, USA) (27). The percentage of β-hexosaminidase release was calculated by dividing the reading from the treatment by the reading from the total.

To determine the inhibitory effect of PTx on MC degranulation, cells were pretreated with PTx (100 ng/mL, 16 h) prior to any stimulation. To determine the inhibitory effect of YM-254890 on MC degranulation, cells were pretreated with YM-254890 (10 μM, 5 min) prior to any stimulation. To determine the inhibitory effect of both PTx and YM-254890 on MC degranulation, cells were pretreated with PTx (100 ng/mL, 16 h) and then washed and incubated with YM-254890 (10 μM, 5 min) prior to any stimulation (28).

To determine receptor desensitization, RBL-MRGPRX2 and LAD2 cells were incubated in the presence or absence of C48/80, CSP-1, and PAMP-12 for 16 h prior to agonist-induced MC degranulation as described above.

For FcϵRI-dependent activation, RBL-2H3 cells were sensitized overnight with anti-DNP IgE and subsequently challenged with DNP-BSA to induce FcϵRI crosslinking.

### ERK1/2 phosphorylation: western blotting

RBL-MRGPRX2 cells were treated with C48/80 (3 µg/mL), CSP-1 (10 µM), PAMP-12 (10 µM), or vehicle control for 2.5 min. Cells were lysed in 1X RIPA buffer with a protease and phosphatase inhibitor cocktail, and the protein lysate was collected and stored at -80 °C immediately till further analysis. The protein concentration was measured using the BCA protein assay kit (Catalog #23227), and 40 µg of total proteins were resolved by SDS-PAGE and subsequently transferred to PVDF membrane. After blocking (5% skim milk in 0.1% Tris-buffered saline, 1 h), blots were then incubated with rabbit anti-pERK1/2 (1:1,000), anti-ERK1/2 (1:1,000), and anti-β-Actin (1:1,000) antibodies at 4 °C overnight. Blots were incubated with specific horseradish peroxidase (HRP)-linked secondary antibodies for 1 h and were developed using Pico plus Chemiluminescence Substrate (Catalog #34577). Images were obtained and analyzed using iBright 1500 Imaging System (Thermo Fisher Scientific).

To determine the inhibitory effect of PTx on ERK1/2 phosphorylation, cells were pretreated with PTx (100 ng/mL, 16 h) prior to any stimulation.

### ERK1/2 phosphorylation: immunofluorescence microscopy

RBL-MRGPRX2 cells (2×10^5^ cells) were plated onto sterilized glass coverslips (12-mm diameter) in a 24-well plate and incubated overnight at 37 °C with 5% CO_2_ to allow for cell adherence. Cells were washed and stimulated with C48/80, CSP-1, PAMP-12, or vehicle control for 2.5 min at 37 °C. Immediately after the treatment, cells were rinsed with ice-cold PBS and fixed with 4% paraformaldehyde for 15 min at 4 °C. The cells were washed with blocking buffer (PBST with 1% BSA) for 1 h at room temperature. Primary antibody incubation was performed using purified unconjugated rabbit anti-phosphorylated ERK1/2 antibody (1:250) at 4 °C overnight. The next day, cells were washed and incubated with AF488-conjugated goat anti-rabbit IgG antibody for 1 h at 4 °C. Then, coverslips with cells were mounted onto the glass slides using a Mounting Medium with DAPI (Fluoroshield, Abcam, Catalog #ab104139), and images were visualized using a Nikon Eclipse Ni microscope.

To determine the inhibitory effect of PTx on ERK1/2 phosphorylation, cells were pretreated with PTx (100 ng/mL, 16 h) prior to any stimulation.

### MRGPRX2 phosphorylation: immunoprecipitation and western blotting

RBL-MRGPRX2 cells (3×10^6^) were plated in a 10-cm sterile Petri dish and incubated overnight in 37 °C with 5% CO_2_ to allow cells adherence. Cells were treated with CSP-1 (10 µM), PAMP-12 (10 µM), or vehicle control for 2.5, 5, and 15 min. Following the treatment, cells were washed twice with PBS and lysed in radioimmunoprecipitation assay buffer (1X RIPA) with a protease and phosphatase inhibitor cocktail, and the protein lysate was collected. Immunoprecipitation was performed according to the Manufacturer’s protocol (Cell Signaling Technology). Briefly, cell lysates were incubated with an anti-HA primary antibody (Biolegend, Clone Poly9023; Catalog #923501) on a rocker at 4 °C overnight. After that, protein A agarose (50% bead slurry, Cell Signaling Technology, Catalog #9863S) was added and incubated for an additional 3–4 h on a rocker at 4 °C. At the end of the incubation period, cell lysates were centrifuged (1000 rpm) for 30 sec at 4 °C, and the pellets were washed and eluted in loading buffer and stored at -80 °C for further analysis.

To determine the MRGPRX2 phosphorylation, 40 µg of immunoprecipitated protein were resolved by SDS-PAGE and subsequently transferred to PVDF membrane. After blocking (5% bovine serum albumin (BSA) in 0.1% Tris-buffered saline, for 1 h), blots were incubated with rabbit anti-pMRGPRX2 (S327/S328 and S313) (1:1,000) and anti-HA (1:1,000) antibodies at 4 °C overnight. Blots were incubated with horseradish peroxidase (HRP)-linked anti-rabbit secondary antibody for 1 h and developed using Femto maximum sensitivity Chemiluminescence Substrate (Catalog #34095). Images were obtained and analyzed using iBright 1500 Imaging System (Thermo Fisher Scientific).

### MRGPRX2 phosphorylation: immunofluorescence microscopy

RBL-MRGPRX2 cells (2×10^5^ cells) were plated onto sterilized glass coverslips (12-mm diameter) in a 24-well plate and incubated overnight at 37 °C with 5% CO_2_ to allow for cell adherence. Cells were washed and stimulated with CSP-1, PAMP-12, or vehicle control for 2.5, 5, and 15 min at 37 °C. Immediately after the treatment, cells were rinsed with ice-cold PBS and fixed with 4% paraformaldehyde for 15 min at 4 °C. The cells were washed with blocking buffer (PBST with 1% BSA) for 1 h at room temperature. Primary antibody incubation was performed using purified unconjugated rabbit anti-phosphorylated MRGPRX2 (S327/S328) antibody (1:250) at 4 °C overnight. The next day, cells were washed and incubated with AF488-conjugated goat anti-rabbit IgG antibody for 1 h at 4 °C. Then, coverslips with cells were mounted onto the glass slides using a Mounting Medium with DAPI (Fluoroshield, Abcam, Catalog #ab104139), and images were visualized using a Nikon Eclipse Ni microscope.

### β-arrestin recruitment: transcriptional activation following arrestin translocation (Tango) assay

HTLA cells are HEK293T cells that stably express β-arrestin2-fused to a TEV protease, a tetracycline controlled transcription factor (tTA) and a luciferase reporter gene. Upon agonist-induced β-arrestin recruitment, TEV-mediated cleavage releases tTA, which subsequently activates a luciferase reporter, generating a quantitative luminescence readout of β-arrestin engagement (24). HTLA cells stably expressing MRGPRX2 (HTLA-MRGPRX2) (5×10^4^ cells) were seeded in a 96-well cell culture plate in triplicates with an antibiotic-free medium and incubated in a 37 °C incubator with 5% CO_2_ for 6 h. After the cells adhered to the bottom of the well, the medium was gently removed, and cells were incubated with C48/80, CSP-1, or PAMP-12 in an antibiotic-free medium for an additional 16 h at 37 °C. Following 16 h incubation, the medium was removed, and Bright-Glo solution (100 μL) was added to each well. The luminescence was measured in a relative luminescence unit (RLU) by Thermo Labsystems Luminoskan Ascent 392 Microplate Luminometer (24).

### Receptor internalization: flow cytometry

RBL-MRGPRX2 or HTLA-MRGPRX2 cells (5×10^5^) were either treated with C48/80, CSP-1, PAMP-12, or vehicle control for 0.5, 3, 6, and 16 h. LAD2 cells (0.3x10^6^) were incubated in the presence or absence of C48/80, CSP-1, and PAMP-12 for 16 h. The cells were then washed and suspended in FACS buffer (PBS containing 2% fetal calf serum (FCS) and 0.09% sodium azide). Cells were incubated with the PE-conjugated anti-MRGPRX2 antibody for 30 min at 4 °C in the dark. Cells were washed with FACS buffer, fixed in 1.5% paraformaldehyde, and were subjected to flow cytometric analysis using a BD LSR II flow cytometer (San Jose, CA, USA). The results were analyzed using WinList software, version 8. Receptor internalization was defined as the loss of cell surface receptor expression (mean fluorescence intensity; MFI) following agonist stimulation when compared with the MFI of vehicle control.

To determine the inhibitory effect of Dyngo-4a on MRGPRX2 internalization, RBL-2H3 cells stably expressing MRGPRX2 were pretreated with Dyngo-4a (30 µM, for 30 min) prior to any stimulation. Note that all experiments with Dyngo-4a were performed with the buffer without albumin or serum (HEPES-containing buffer without BSA and FACS buffer without FCS).

To determine the inhibitory effect of cycloheximide on cell surface MRGPRX2 expression, cells were treated with cycloheximide (5 µg/mL) together with any stimulation.

### Receptor internalization: immunofluorescence microscopy

Direct visualization of internalized receptors induced by C48/80, CSP-1, and PAMP-12 was evaluated according to the previously published protocol (23). Briefly, RBL-MRGPRX2 cells (2×10^5^ cells) were plated onto sterilized glass coverslips (12-mm diameter) in a 24-well plate and incubated overnight at 37 °C with 5% CO_2_ to allow for cell adherence. Cells were rinsed with PBS and blocked with blocking buffer (PBS with 2% BSA) for 30 min at room temperature. Primary antibody incubation was performed using purified unconjugated mouse anti-MRGPRX2 antibody (1:250) for 1 h at 4 °C to label the cell surface expressed receptors. Cells were then washed and stimulated with C48/80, CSP-1, or PAMP-12 for 30 min at 37 °C to allow for internalization. Next, cells were fixed with 4% paraformaldehyde for 15 min at 4 °C. Labeled surface receptors were detected by incubating the cells with a saturated concentration of AF647-conjugated anti-mouse IgG antibody (red) for 1 h at 4 °C. Cells were washed vigorously to remove excess antibody, then permeabilized using 0.2% Triton X-100 in blocking buffer for 30 min. The internalized receptors were detected by incubating cells with AF488-conjugated anti-mouse antibody (green) for 1 h at 4 °C. Then, coverslips with cells were mounted onto the glass slides using a ProLong Gold Antifade mounting medium (Invitrogen), and images were visualized using a Nikon Eclipse Ni microscope.

To determine the inhibitory effect of Dyngo-4a on MRGPRX2 internalization, cells were pretreated with Dyngo-4a (30 µM, 30 min) prior to any stimulation as described above.

### Cell viability assay

Cell viability was determined using a 3-(4,5-dimethylthiazol-2-yl)-2,5-diphenyltetrazolium bromide (MTT) assay. Briefly, RBL-MRGPRX2 cells were seeded in a 96-well plate (5×10^4^ cells/well) in 200 μL volume of DMEM supplemented with 10% FBS and incubated overnight at 37 °C to allow for cell adherence. The following day, the medium was removed and replaced with a fresh serum-free medium containing C48/80 (10 μg/mL), CSP-1 (10 µM), PAMP-12 (10 µM), or vehicle control. Following a 16 h incubation at 37 °C, the solutions were removed, replaced with MTT solution (0.45 µg/mL), and further incubated for 4 h at 37 °C. The cells’ viability was determined by dissolving the formazan product produced by treated cells in dimethyl sulfoxide (DMSO) and measuring absorbance at 570 nm using a Versamax microplate spectrophotometer (Molecular Devices, San Jose, CA, USA). The percentage of cell viability was calculated by dividing the reading from the treatment group by the reading from the vehicle control.

### Total receptor expression: flow cytometry

RBL-MRGPRX2 cells (1.5×10^6^) were either treated with C48/80 (3 µg/mL), CSP-1 (10 µM), PAMP-12 (10 µM), or vehicle control for 16 h with or without cycloheximide co-treatment (5 µg/mL) (29, 30). Cells were then washed and suspended in FACS buffer (PBS containing 2% fetal calf serum (FCS) and 0.09% sodium azide), fixed with 1.5% paraformaldehyde for 20 min at room temperature, and washed with 1X permeabilization buffer (BioLegend). Cells were then resuspended in 1X permeabilization buffer and incubated with the PE-conjugated anti-MRGPRX2 antibody for 30 min at 4 °C in the dark, washed with FACS buffer, and were subjected to flow cytometric analysis using a BD LSR II flow cytometer (San Jose, CA, USA). The results were analyzed using WinList software, version 8. The total receptor expression was determined using the mean fluorescent intensity (MFI).

### Total receptor expression: western blotting

RBL-MRGPRX2 (HA-tagged; 1.5-2×10^6^) cells were treated with C48/80 (3 µg/mL), CSP-1 (10 µM), PAMP-12 (10 µM), or vehicle control for 16 h with or without cycloheximide co-treatment (5 µg/mL) (29, 30). Cells were lysed in 1X RIPA buffer with a protease inhibitor cocktail, and the protein lysate was collected and stored at -80 °C for further analysis. The protein concentration was measured using the BCA protein assay kit (Catalog #23227) and 40 µg of total proteins resolved by SDS-PAGE and subsequently transferred to PVDF membrane. After blocking (5% skim milk in 0.1% Tris-buffered saline, 1 h), blots were incubated with mouse anti-HA (0.4 µg/mL) and anti-β-Actin (1:1,000) antibodies at 4 °C overnight. Blots were incubated with specific horseradish peroxidase (HRP)-linked secondary antibodies for 1 h and were developed using Femto maximum sensitivity Chemiluminescence Substrate (Catalog #34095). Images were obtained and analyzed using iBright 1500 Imaging System (Thermo Fisher Scientific).

### Statistical analysis

Data shown are mean ± SEM values derived from at least three independent experiments. Statistical significance was determined by one-way or two-way ANOVA, as indicated. Significant differences were set at *p≤ 0.05, **p≤ 0.01, ***p≤ 0.001, ****p≤ 0.0001 and analyzed by GraphPad Prism version 9.0.1.

## Results

### MC activation via MRGPRX2 by C48/80, CSP-1, and PAMP-12 is mediated by Gαq, Gαi1, and Gαi3, but not Gαi2

**Figure 1:**
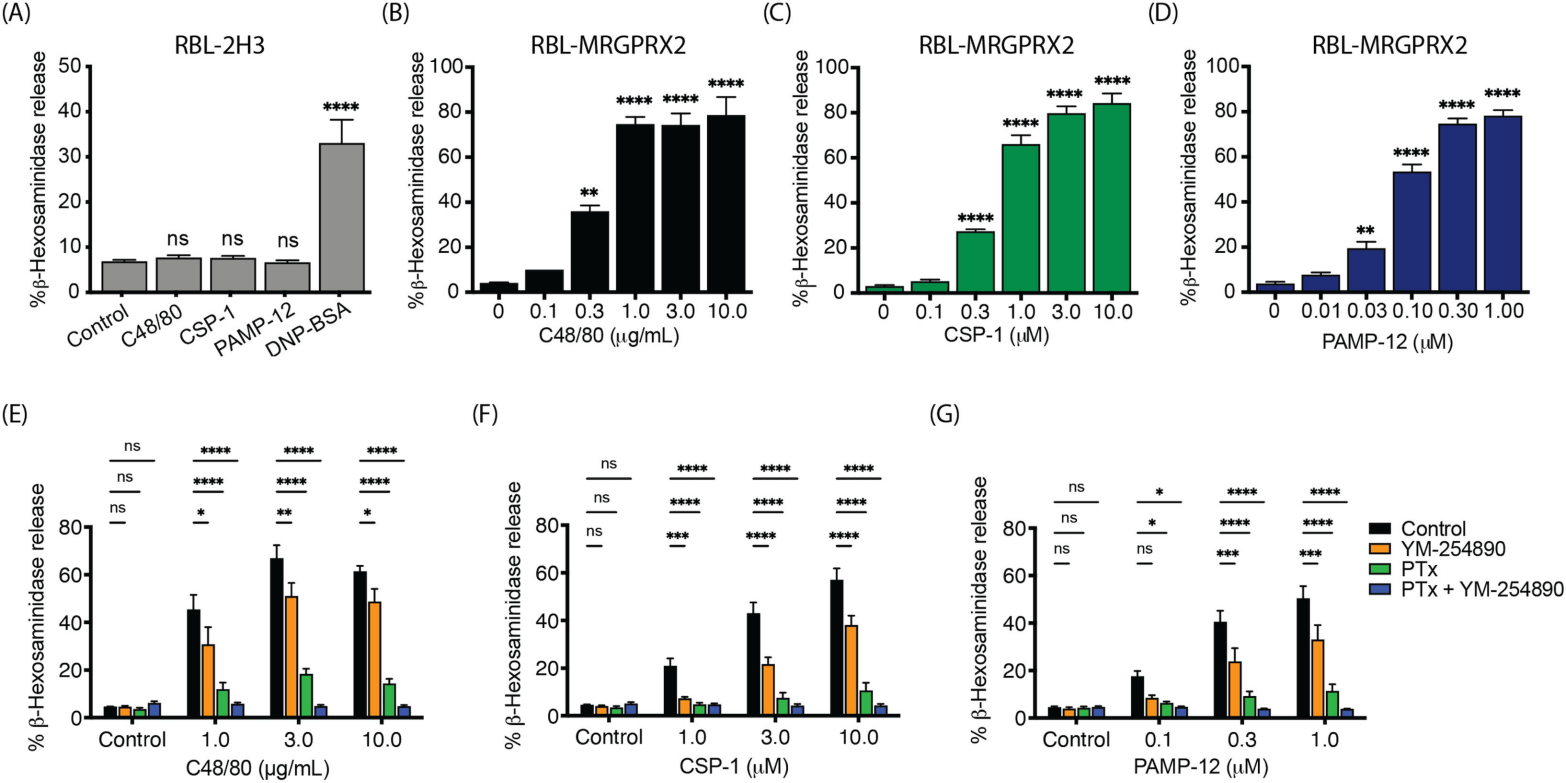
MRGPRX2-dependent MC activation by C48/80, CSP-1, and PAMP-12 is mediated by G proteins. **(A)** RBL-2H3 cells were stimulated with C48/80, CSP-1, PAMP-12, and DNP-BSA/IgE (positive control) for 30 min and β-hexosaminidase release was determined. RBL-MRGPRX2 cells were stimulated with indicated concentrations of **(B)** C48/80, **(C)** CSP-1, and **(D)** PAMP-12 for 30 min, and β-hexosaminidase release was determined. RBL-MRGPRX2 cells were incubated with or without pertussis toxin (PTx, 100 ng/mL, for 16 h) and washed and incubated with or without YM-254890 (10 μM, for 5 min) and then stimulated with indicated concentrations of **(E)** C48/80, **(F)** CSP-1, and **(G)** for 30 min, and β-hexosaminidase release was determined. Data are the mean ± SEM of n = 3 independent experiments. Statistical significance was determined by One-way ANOVA and Dunnett’s multiple comparisons at a value * p < 0.05, ** p < 0.01, *** p < 0.001, **** p < 0.0001, and ns denotes “not significant”.

Previous studies demonstrated that CSP-1 and PAMP-12 induce degranulation in a human MC line, LAD2 cells that endogenously express MRGPRX2 (7, 31). We first sought to determine if these agonists induce MC degranulation via MRGPRX2 using RBL-2H3 cells, a rodent MC line that does not express MRGPRX2. We used C48/80, a classic MRGPRX2 agonist, as a control. While these cells did not degranulate when exposed to any of the agonists tested, they responded robustly to antigen (DNP-BSA) stimulation through FcϵRI crosslinking of anti-DNP IgE-sensitized receptors. (**Figure 1A**). However, when RBL-2H3 cells stably expressing MRGPRX2 (RBL-MRGPRX2) were used, they responded to both CSP-1 and PAMP-12 for degranulation with EC_50_ values (half-maximal effective concentration) of 0.45 µM and 0.07 µM, respectively. Despite this difference, both peptides and C48/80 induced similar maximal responses (**Figures 1B–D**). These findings indicate that CSP-1 and PAMP-12 trigger MC degranulation via MRGPRX2. Cryo-electron analysis of MRGPRX2 structure revealed that upon agonist stimulation, the receptor activates both Gαi and Gαq pathways (32, 33). However, in human MCs, Gαi family of G proteins is more important for MRGPRX2-mediated degranulation than Gαq. To determine the role of Gα family of G proteins on MRGPRX2-mediated degranulation in RBL-MRGPRX2 cells, we utilized a pharmacological approach using a Gαi-specific inhibitor (Pertussis toxin; PTx) and a Gαq-specific inhibitor (YM-254890) and demonstrated that C48/80, CSP-1, and PAMP-12-induced degranulation were partially inhibited following YM-254890 pretreatment (10 µM, for 5 min), but substantially inhibited following PTx pretreatment (100 ng/mL, for 16 h) (**Figures 1E–G**). These findings suggest that as for primary human MCs, MRGPRX2 predominantly utilizes Gαi family of G proteins for degranulation in transfected RBL-MRGPRX2 cells.

**Figure 2:**
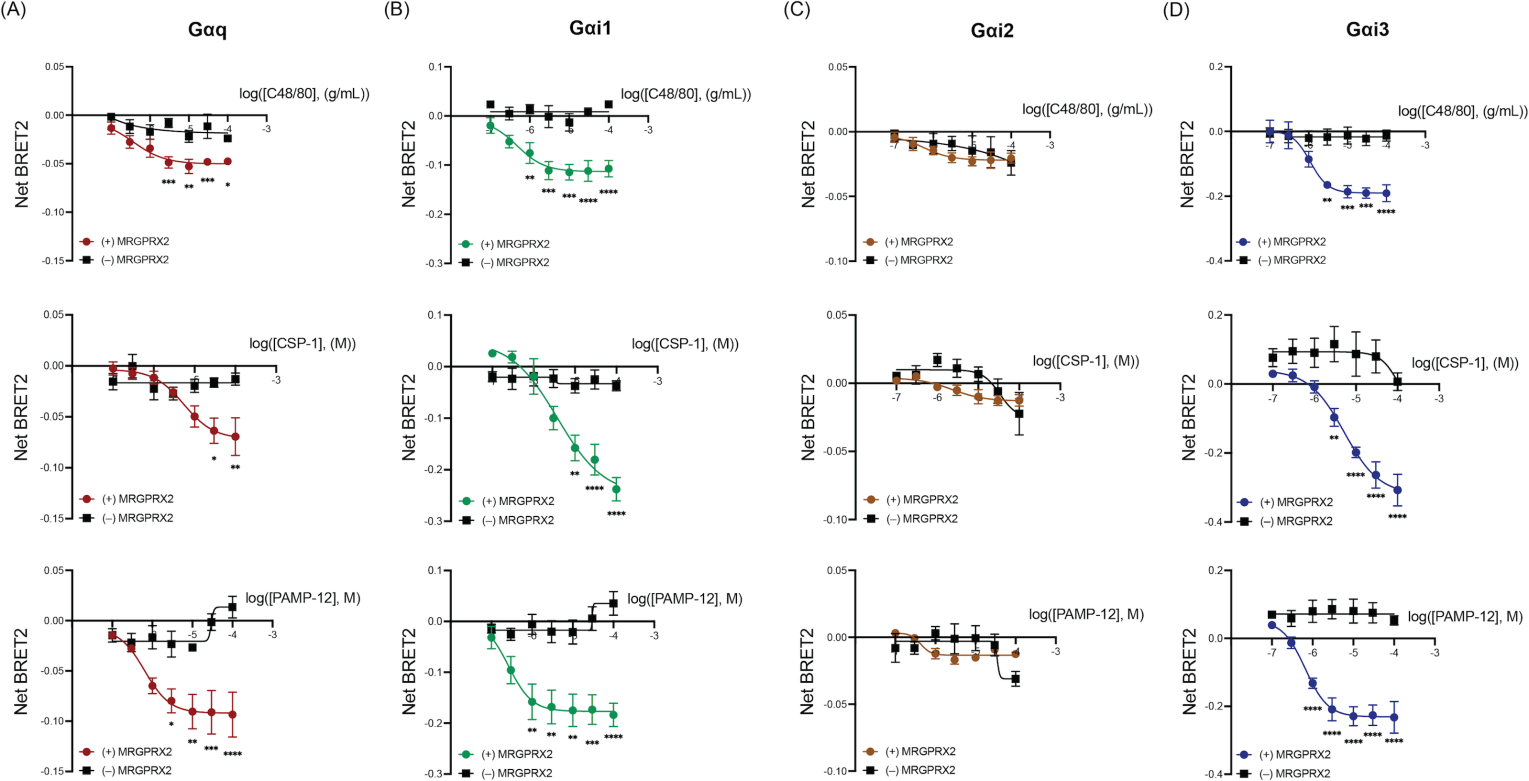
MRGPRX2-dependent MC activation by C48/80, CSP-1, and PAMP-12 is mediated by Gαq, Gαi1, and Gαi3, but not Gαi2. HEK-293T cells transfected with Gβ, Gγ-GFP2, and **(A)** Gαq-RLuc8, **(B)** Gαi1-RLuc8, or **(C)** Gαi2-RLuc8, or **(D)** Gαi3-RLuc8 were stimulated with indicated concentrations of C48/80, CSP-1, and PAMP-12. Net BRET2 signals were calculated in comparison to vehicle-treated control. Data are the mean ± SEM of n = 3–5 independent experiments. Statistical significance was determined by Two-way ANOVA and Šídák’s multiple comparisons at a value * p < 0.05, ** p < 0.01, *** p < 0.001, and **** p < 0.0001.

To determine the G protein coupling specificity of MRGPRX2, we utilized the newly developed “TRUPATH” bioluminescence resonance energy transfer 2 (BRET2) biosensors, which have been developed to interrogate signal transducers with single pathway resolution in cells (26, 34). We transfected HEK293T cells with cDNA encoding MRGPRX2 and the G protein BRET2 sensors. Transfected cells were exposed to different concentrations of MRGPRX2 agonists, and BRET2 ratios were measured. Interestingly, we found that C48/80, CSP-1, and PAMP-12 coupled to Gαq, Gαi1, and Gαi3, but not Gαi2 (**Figures 2A–D**). These findings demonstrate that all MRGPRX2 agonists tested transduce similar G protein signaling despite their distinct biological functions.

### The phosphorylation of ERK1/2 following MRGPRX2 activation in response to C48/80, CSP-1, and PAMP-12 is similar

**Figure 3:**
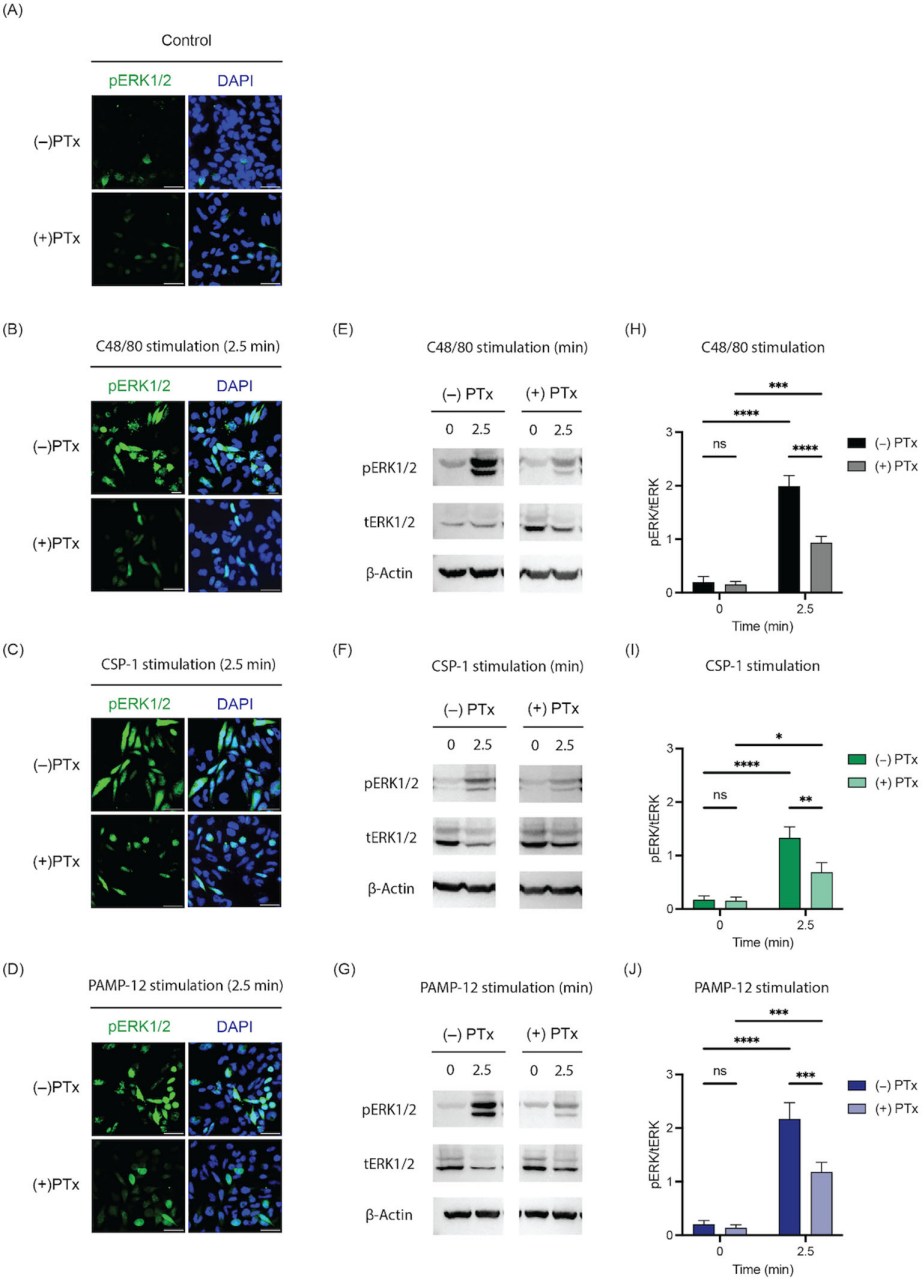
The phosphorylation of ERK1/2 following MRGPRX2 activation in response to C48/80, CSP-1, and PAMP-12 is similar. RBL-MRGPRX2 cells were preincubated with or without pertussis toxin (PTx, 100 ng/mL, for 16 h) and then exposed to vehicle control, C48/80 (3 µg/mL), CSP-1 (10 µM), and PAMP-12 (10 µM) for 2.5 min. **(A–D)** Immunofluorescence staining of phosphorylated ERK1/2. Representative images were shown. Scale bar 50 px = 10 μm. **(E–G)** The treated cells were lysed, cell lysates were collected, and western blotting was performed with anti-pERK1/2, anti-ERK1/2, and anti-β-actin antibodies. Representative blots were shown. **(H–J)** Bar graphs represent the relative ratios of phosphorylated ERK1/2 (pERK1/2) over total ERK1/2 (tERK1/2). Data presented are the mean ± SEM of n = 6–8 independent experiments. Statistical significance was determined by Two-way ANOVA and Tukey’s multiple comparisons at a value * p < 0.05, ** p < 0.01, *** p < 0.001, **** p < 0.0001, and ns denotes “not significant”.

Previous studies have demonstrated that C48/80, SP, and codeine activate MRGPRX2, leading to degranulation that is dependent on the ERK1/2 phosphorylation in LAD2 and MRGPRX2 cells (35–37). To determine whether CSP-1 and PAMP-12 induce similar ERK1/2 phosphorylation kinetics, RBL-MRGPRX2 cells were stimulated with these agonists for 0 and 2.5 min, and ERK1/2 phosphorylation was assessed by immunofluorescence staining using C48/80 as a positive control. The cells were incubated with an unconjugated rabbit anti-phospho-ERK1/2 primary antibody, followed by Alexa Fluor™ (AF) 488-conjugated anti-rabbit IgG to visualize phosphorylated ERK1/2 within the treated cells. To assess the role of Gαi signaling, cells were pretreated with pertussis toxin (PTx) or vehicle control prior to stimulation with MRGPRX2 agonists. As shown in **Figures 3A–D**, cells stimulated for 2.5 min with C48/80, CSP-1, or PAMP-12 exhibited strong phosphorylated ERK1/2 immunofluorescence, which was markedly reduced by PTx pretreatment.

To validate this finding, we performed a Western blotting experiment of RBL-MRGPRX2 cells treated as described above. Both CSP-1 and PAMP-12 caused a significant increase in ERK1/2 phosphorylation, similar to C48/80-treated cells but with differing magnitudes of response (**Figures 3E–J**). To determine the contribution of Gαi signaling, RBL-MRGPRX2 cells were pretreated with PTx (100 ng/mL, for 16 h) and exposed to CSP-1 or PAMP-12. Confirming the results obtained from immunofluorescence staining, the PTx pretreatment markedly reduced ERK1/2 phosphorylation in response to both CSP-1 and PAMP-12, indicating that ERK1/2 activation is predominantly Gαi-dependent. These findings, together with the G protein coupling specificity study above, suggest that both CSP-1 and PAMP-12 are similar in their ability to induce MRGPRX2 activation via the G protein pathway. However, given that β-arrestin-mediated signaling has also been implicated in GPCR activation (38, 39), we speculate that CSP-1 and PAMP-12 may differ in their ability to recruit β-arrestin following MRGPRX2 activation.

### CSP-1 induces minimal phosphorylation at Ser327, Ser328, and Ser313 of MRGPRX2, whereas PAMP-12 elicits robust phosphorylation at these residues

**Figure 4:**
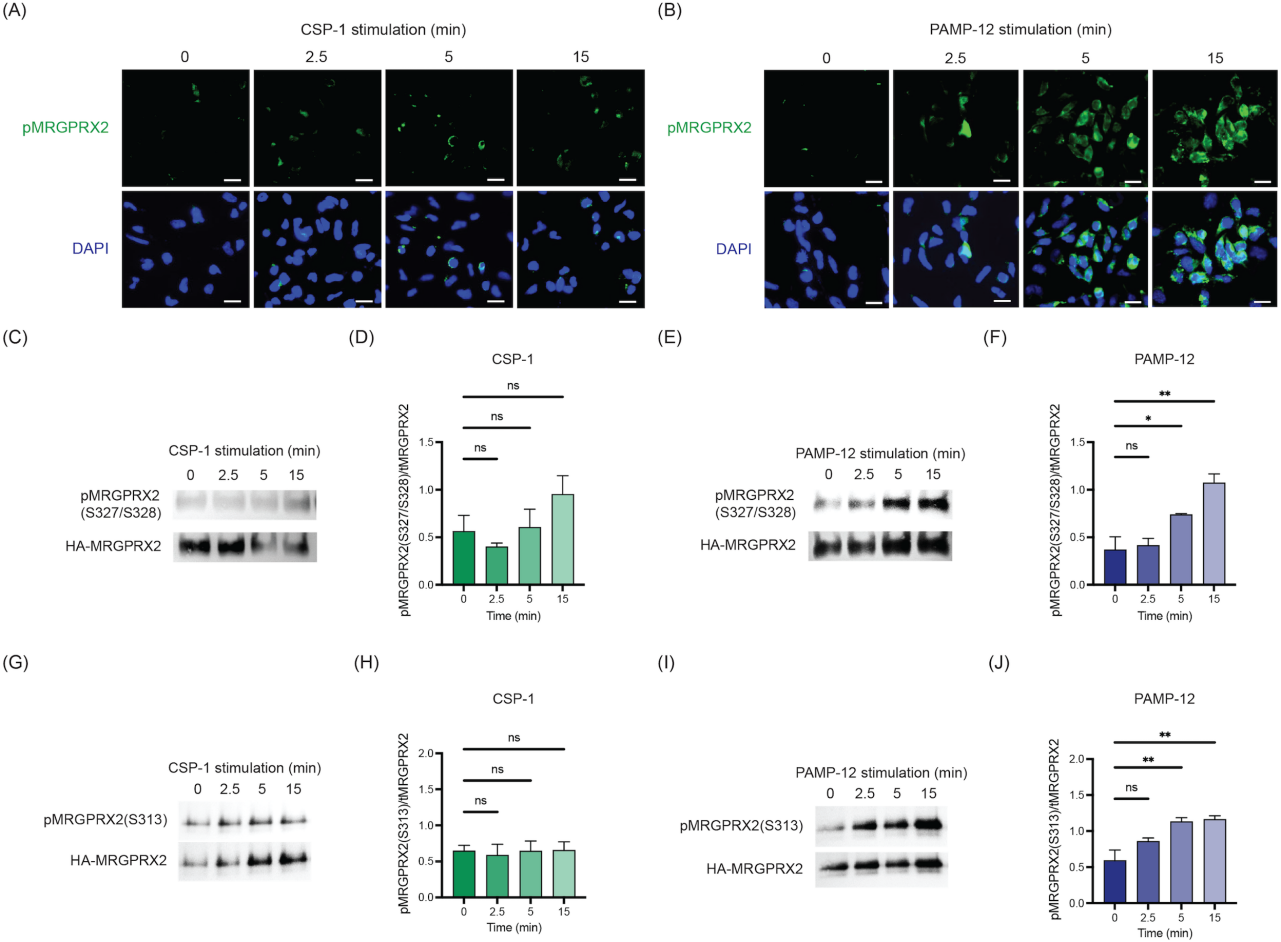
CSP-1 induces minimal phosphorylation at Ser327, Ser328, and Ser313 of MRGPRX2, whereas PAMP-12 elicits robust phosphorylation at these residues. RBL-MRGPRX2 cells were incubated with CSP-1, PAMP-12, or vehicle for 2.5, 5, and 15 min. **(A, B)** Immunofluorescence staining of phosphorylated Ser327 and Ser328 of MRGPRX2. Representative images were shown. Scale bar = 50 μm. **(C, E, G, I)** Following stimulation, cells were lysed, and MRGPRX2-HA was immunoprecipitated using an anti-HA antibody. Western blotting was performed using phospho-specific antibodies against pMRGPRX2 (Ser327/Ser328) **(C, E)** or pMRGPRX2 (Ser313) (G and I), with anti-HA used to detect total receptor levels. Representative blots are shown. **(D, F, H, J)** Bar graphs represent the relative ratios of phosphorylated MRGPRX2 (pMRGPRX2) over total MRGPRX2 (tMRGPRX2 or HA-MRGPRX2). Data presented are the mean ± SEM of n = 3–4 independent experiments. Statistical significance was determined by One-way ANOVA and Dunnett’s multiple comparisons at a value * p < 0.05, ** p < 0.01, and ns denotes “not significant”.

It is well-documented that the phosphorylation of GPCRs usually precedes β-arrestin binding and recruitment (40, 41). MRGPRX2 has five potential phosphorylation sites within its carboxyl-terminal tail, located at Ser313, Thr321, Ser325, Ser327, and Ser328 (28). Phosphorylation-specific antibodies against Ser327/Ser328 and Ser313 were recently developed because the phosphorylation at these sites likely result in the interaction of the receptor with β-arrestin. To determine if MRGPRX2 undergoes phosphorylation at Ser327/328, RBL-MRGPRX2 cells were stimulated with CSP-1 or PAMP-12 for 0, 2.5, 5, and 15 min, and phosphorylation was assessed by immunofluorescence staining using anti-phospho-Ser327/328-specific antibody. We found that CSP-1 caused little to no phosphorylation, but PAMP-12 induced a robust response (**Figures 4A, B**).

To quantify these signals, we performed Western blot analysis on RBL-MRGPRX2 cells treated as described above. MRGPRX2 was immunoprecipitated using an anti-HA antibody, as the receptor construct contains an HA epitope tag. Forty micrograms of immunoprecipitated MRGPRX2-HA were resolved by SDS-PAGE and probed with anti-phospho-Ser327/Ser328 and anti-HA antibodies to assess receptor phosphorylation and total receptor, respectively. Consistent with the immunofluorescence findings, CSP-1-treated cells exhibited weak phosphorylation at Ser327/Ser328 with a trend toward increased signal intensity following prolonged agonist exposure; however, this increase did not reach statistical significance (**Figures 4C, D**). By contrast, PAMP-12 treatment resulted in robust phosphorylation at these residues (**Figures 4E, F**). Using the antibody specific for Ser313, we found that CSP-1 did not induce MRGPRX2 phosphorylation at this site, but PAMP-12 caused a significant response at 5 and 15 min after stimulation (**Figures 4G–J**).

### CSP-1 is a G protein-biased, whereas PAMP-12 is a balanced agonist for MRGPRX2

**Figure 5:**
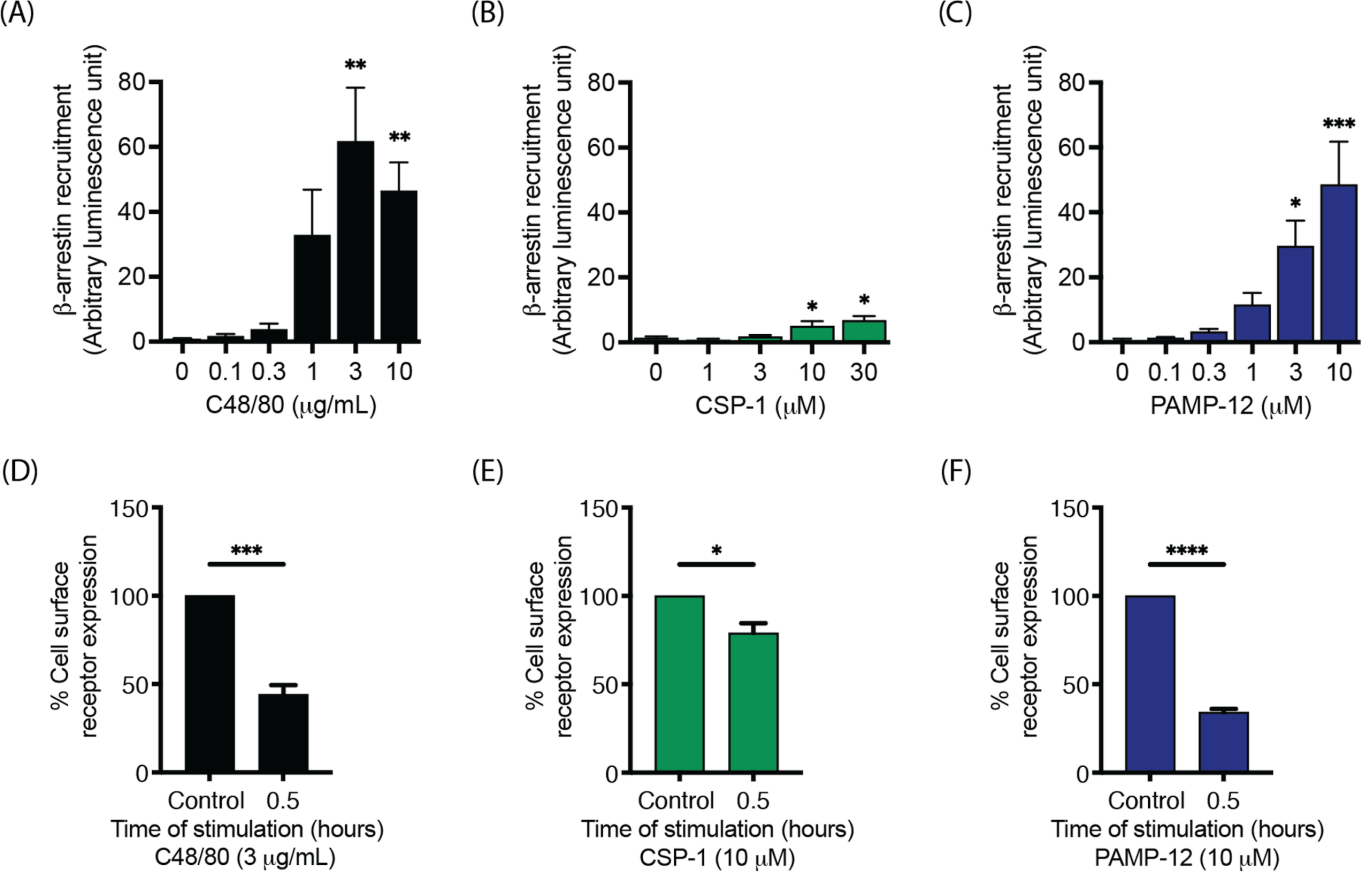
CSP-1 is a G protein-biased, whereas PAMP-12 is a balanced agonist for MRGPRX2. HTLA-MRGPRX2 cells were incubated with indicated concentrations of **(A)** C48/80, **(B)** CSP-1, and **(C)** PAMP-12 for 16 h. The medium was removed and replaced with 100 µL per well of Bright-Glo solution. The β-arrestin recruitment (in relative luminescence unit) was measured. HTLA-MRGPRX2 cells were incubated with **(D)** C48/80, **(E)** CSP-1, and **(F)** PAMP-12 for 0.5 h, and the cell surface receptor expression was determined by flow cytometry and quantitated using a mean fluorescent intensity (MFI) in comparison to the vehicle-treated control. Data are the mean ± SEM of n = 3–4 independent experiments. Statistical significance was determined by One-way ANOVA and Dunnett’s multiple comparisons (A-C) and unpaired T test (D-F) at a value * p < 0.05, ** p < 0.01, *** p < 0.001, and **** p < 0.0001.

It has previously been demonstrated that certain MRGPRX2 agonists, i.e., C48/80, substance P (SP), and codeine, activate the β-arrestin pathway by recruiting β-arrestins to the activated receptor and causing receptor internalization (20, 23, 42). By contrast, the angiogenic host defense peptide (HDP) AG-30/5C and the HDP mimetic murepavadin do not (20, 21). To determine if CSP-1 and PAMP-12 activate the β-arrestin signaling pathway, we utilized HTLA-MRGPRX2 cells and TANGO assay in which transcriptional activation of the luciferase reporter gene is only possible when β-arrestin is recruited to the activated receptor. We used C48/80 as a positive control. Interestingly, concentrations of each of these agonists that induced half maximal degranulation in RBL-MRGPRX2 cells did not induce β-arrestin recruitment in HTLA-MRGPRX2 cells (**Figures 5A–C**). However, at concentrations ≥10-fold higher than those required for degranulation, C48/80 (10 µg/mL) and PAMP-12 (10 µM) induced an approximately 50-fold increase in luminescence (indicative of β-arrestin recruitment and activation), whereas CSP-1 (10 µM and 30 µM) induced only 5-fold increase (**Figures 5A–C**). These results indicate that, in addition to G protein signaling, PAMP-12 also strongly activates the β-arrestin pathway, whereas CSP-1 does not.

To determine if β-arrestin recruitment is associated with receptor internalization, we assessed cell surface expression of the receptor following stimulation of HTLA-MRGPRX2 cells with CSP-1 (10 µM) and PAMP-12 (10 µM) using cells treated with C48/80 (10 µg/mL) as positive controls. Cells were exposed to MRGPRX2 agonists or vehicle control for 30 min and cell surface receptor expression was assessed using PE-conjugated anti-MRGPRX2 antibody. Notably, cells treated with CSP-1 resulted in a reduction in cell surface MRGPRX2 expression by ~15-20%, while those treated with PAMP-12 exhibited a substantial reduction in cell surface receptor expression (~60%) similar to C48/80 control (**Figures 5D–F**). Taken together, these findings suggest that CSP-1 preferentially activates MRGPRX2 via the G protein pathway, whereas PAMP-12 activates MRGPRX2 via both the G protein and the β-arrestin pathways.

### MRGPRX2 internalization in response to C48/80, CSP-1, and PAMP-12 is sensitive to dynamin inhibition

**Figure 6:**
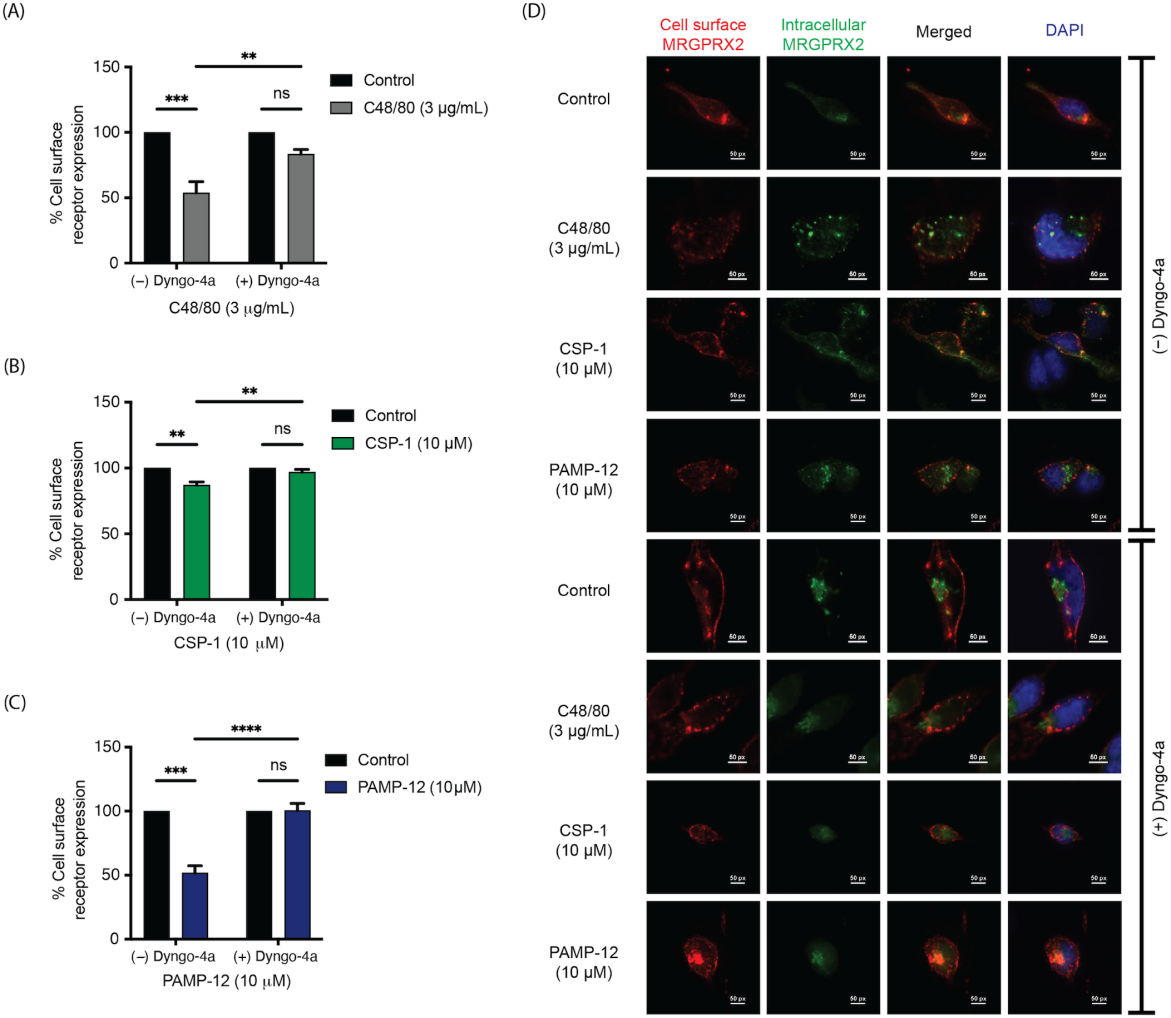
MRGPRX2 internalization in response to C48/80, CSP-1, and PAMP-12 is sensitive to dynamin inhibition. RBL-MRGPRX2 cells were incubated with or without Dyngo-4a (30 μM, for 30 min) and stimulated with **(A)** C48/80, **(B)** CSP-1, and **(C)** PAMP-12 for an additional 30 min. The cell surface receptor expression was determined by flow cytometry and quantitated using a mean fluorescent intensity (MFI) in comparison to the vehicle-treated control. Data are the mean ± SEM of n = 3 independent experiments. Statistical significance was determined by Two-way ANOVA and Tukey’s multiple comparisons at a value ** p < 0.01, *** p < 0.001, **** p < 0.0001, and ns denotes “not significant”. **(D)** Immunofluorescence microscopic analysis of cell surface and internalized MRGPRX2 receptor in RBL-MRGPRX2. Cells were preincubated with or without Dyngo-4a (30 μM, 30 min) and stimulated with C48/80, CSP-1, and PAMP-12 for an additional 30 min. Scale bar 50 px = 10 mm.

We have recently shown that cell surface MRGPRX2 undergoes internalization via both endocytosis and macropinocytosis in response to SP (43). However, the possibility that different MRGPRX2 agonists induce receptor internalization differently has not been investigated. To determine the mode of MRGPRX2 internalization, RBL-MRGPRX2 cells were pretreated with Dyngo-4a, an inhibitor of dynamin GTPase, and receptor internalization in response to C48/80, CSP-1, and PAMP-12 were determined. As shown in **Figures 6A–C**, a 30-min exposure of RBL-MRGPRX2 cells to C48/80, CSP-1, and PAMP-12 provoked a decrease in cell surface receptor expression that is consistent with the results found in HTLA-MRGPRX2 cells (**Figures 5D–F**). Interestingly, Dyngo-4a pretreatment resulted in the inhibition of MRGPRX2 internalization in response to all three agonists tested (**Figures 6A–C**).

To visualize the internalized receptor following exposure of RBL-MRGPRX2 cells to these agonists, we performed an immunofluorescence study as previously described (23). First, we labeled the cell surface receptors with an unconjugated mouse anti-MRGPRX2 primary antibody. Next, cells were exposed to MRGPRX2 agonists to induce receptor internalization. Then, the cells were fixed, and the remaining cell surface receptors were labeled with AF647-conjugated anti-mouse IgG (red). Lastly, the cells were permeabilized, and the pre-labeled internalized receptors were labeled with AF488-conjugated anti-mouse IgG (green). To inhibit dynamin GTPase function, the cells were pretreated with Dyngo-4a or vehicle control prior to MRGPRX2 agonist exposure. As shown in **Figure 6D**, the cell surface MRGPRX2 underwent internalization and demonstrated a punctate pattern in response to C48/80, CSP-1, and PAMP-12. However, despite the retained punctate pattern, the MRGPRX2 remained on the cell surface in cells pretreated with Dyngo-4a. Taken together, these findings suggest that C48/80-, CSP-1-, and PAMP-12-induced MRGPRX2 internalization involves a Dyngo-4a-sensitive, dynamin-associated endocytic pathway.

### PAMP-12 desensitizes and cross-desensitizes the MRGPRX2 to attenuate MC activation

**Figure 7:**
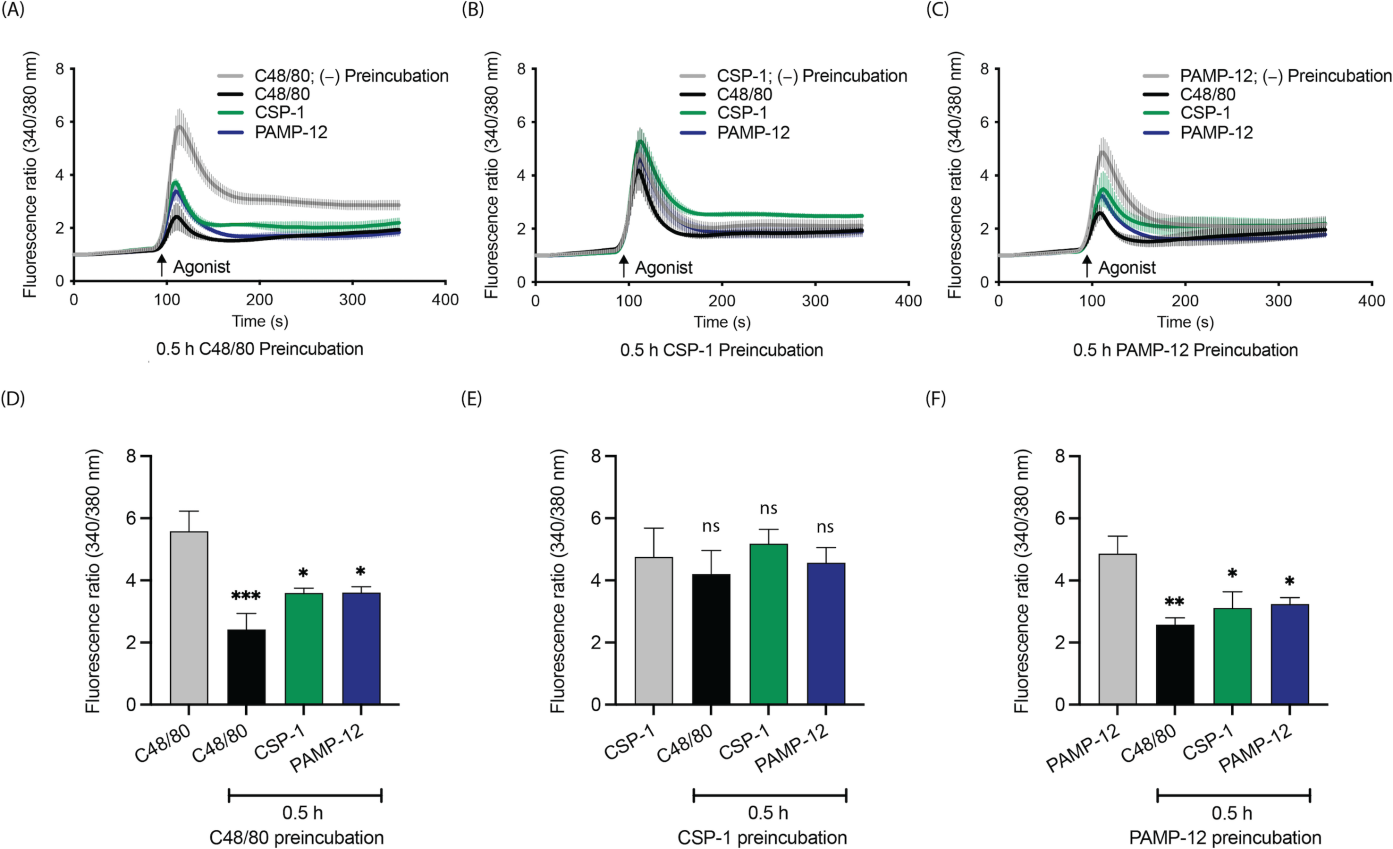
PAMP-12 desensitizes and cross-desensitizes the MRGPRX2 to attenuate MC activation. RBL-MRGPRX2 cells were preincubated with **(A)** C48/80 (3 µg/mL), **(B)** CSP-1 (10 µM), and **(C)** PAMP-12 (10 µM) for 0.5 h and were stimulated again with C48/80 (3 µg/mL; black line), CSP-1 (10 µM; green line), and PAMP-12 (10 µM; blue line). Calcium mobilization was determined by measuring the fluorescence ratios. **(D–F)** The peak calcium responses were plotted as bar graphs for statistical analyses. Data are the mean ± SEM of n = 3–4 independent experiments. Statistical significance was determined by Two-way ANOVA and Dunnett’s multiple comparisons at a value * p < 0.05, ** p < 0.01, *** p < 0.001, and ns denotes “not significant”.

Receptor desensitization is an important mechanism that attenuates GPCR responsiveness to subsequent activation. To determine if CSP-1 and PAMP-12 desensitize and cross-desensitize each other, we performed calcium mobilization assays in Fura-2 AM-loaded RBL-MRGPRX2 cells using C48/80 as a positive control. Cells were stimulated with agonists, and the changes in fluorescence ratio, indicative of calcium signaling were measured. We found that cells preincubated with C48/80 (3 µg/mL, for 0.5 h) and PAMP-12 (10 µM, for 0.5 h) showed a substantial inhibition of calcium mobilization responses to a second stimulation by all agonists (**Figures 7A, C, D, F**). On the contrary, cells preincubated with CSP-1 (10 µM, 0.5 h) responded similarly to those without preincubation regardless of the agonists used (**Figures 7B, E**). These results suggest that PAMP-12 desensitizes and cross-desensitizes the receptor to limit further MC activation, whereas CSP-1 does not.

### MRGPRX2 responsiveness differs after prolonged agonist exposure

**Figure 8:**
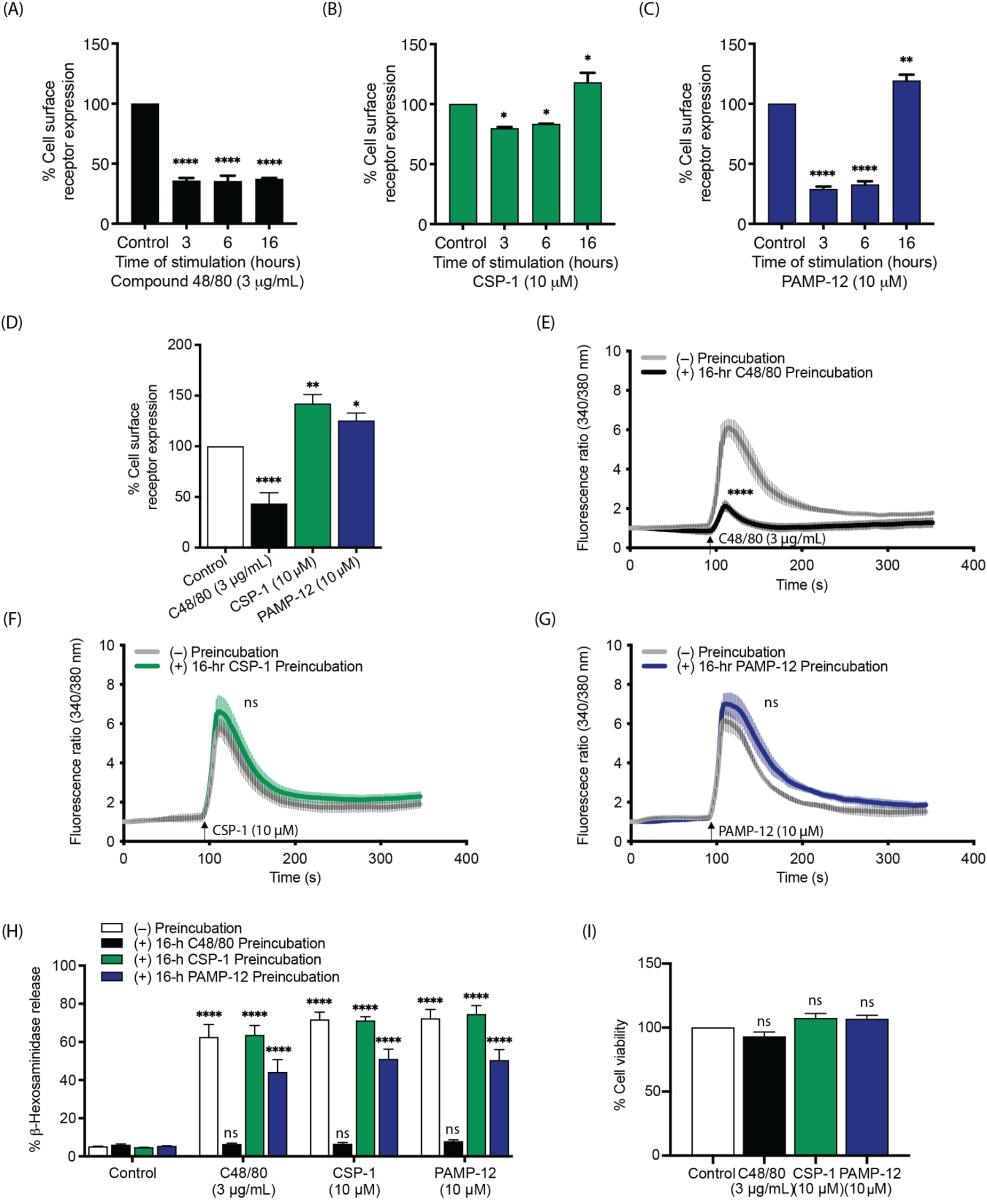
MRGPRX2 responsiveness differs after prolonged agonist exposure. HTLA-MRGPRX2 cells were incubated with **(A)** C48/80, **(B)** CSP-1, and **(C)** PAMP-12 for 3, 6, and 16 h, then the cell surface receptor expression was determined by flow cytometry and quantitated using mean fluorescent intensity (MFI) in comparison to the vehicle-treated control. **(D–I)** RBL-MRGPRX2 cells were preincubated with C48/80 (3 µg/mL), CSP-1 (10 µM), PAMP-12 (10 µM), and vehicle control for 16 h. **(D)** The cell surface MRGPRX2 expression was determined by flow cytometry and quantitated using a mean fluorescent intensity (MFI) in comparison to the vehicle-treated control. **(E–G)** Preincubated cells were stimulated again with the same respective agonist; **(E)** C48/80 (3 µg/mL), **(F)** CSP-1 (10 µM), and **(G)** PAMP-12 (10 µM) and calcium mobilization were determined by measuring the fluorescence ratios. **(H)** Preincubated cells were stimulated with C48/80 (3 µg/mL), CSP-1 (10 µM), and PAMP-12 (10 µM) for an additional 30 min, and β-hexosaminidase release was determined. **(I)** The cell viability of the preincubated cells was determined using an MTT assay. Data are the mean ± SEM of n = 3 independent experiments. Statistical significance was determined by One-way ANOVA and Dunnett’s multiple comparisons (for **D, I**) or Two-way ANOVA and Dunnett’s multiple comparisons (for **H**) or Two-way ANOVA and Šídák’s multiple comparisons (for **E–G**) at a value * p < 0.05, ** p < 0.01, **** p < 0.0001, and ns denotes “not significant”.

The data presented in **Figures 5** and **6** above demonstrated that there are differences in the magnitude of receptor internalization measured at 30 min after stimulation based on the agonist used for MRGPRX2 activation. Herein, we sought to determine the fate of MRGPRX2 in response to different agonists at longer time points after stimulation. For this, HTLA-MRGPRX2 cells were incubated with C48/80, CSP-1, and PAMP-12 for 3, 6, and 16 h, and cell surface receptor expression was determined by flow cytometry. As shown in **Figure 8A**, C48/80 induced a substantial reduction of cell surface MRGPRX2 at 3 h, which remained largely unchanged at 6 and 16 h. However, when cells were exposed to CSP-1 and PAMP-12 for 16 h, they showed significantly greater cell surface receptor expression than untreated controls (**Figures 8B, C**). These findings indicate that prolonged exposure to distinct agonists results in different patterns of MRGPRX2 surface expression over time.

To determine whether the increased cell surface MRGPRX2 observed following CSP-1 and PAMP-12 treatment was associated with altered cellular responsiveness, we first validated receptor expression in RBL-MRGPRX2 cells following an overnight (16 h) treatment with different MRGPRX2 agonists. Like HTLA-MRGPRX2 cells, C48/80 induced a substantial reduction in cell surface receptor expression in RBL-MRGPRX2 cells, whereas CSP-1 and PAMP-12 did not (**Figure 8D**). Next, we utilized a calcium mobilization assay to evaluate the functional responses of cells following prolonged agonist exposure. RBL-MRGPRX2 cells preincubated with C48/80 (3 µg/mL for 16 h) displayed a marked reduction in calcium influx upon subsequent stimulation with the same agonist (**Figure 8E**). In contrast, cells preincubated with CSP-1 (10 µM) or PAMP-12 (10 µM) exhibited calcium responses comparable to those of control cells (**Figures 8F, G**). These results suggest that prolonged exposure to CSP-1 or PAMP-12 does not impair the ability of the cells to respond to further MRGPRX2 activation, whereas extended exposure to C48/80 leads to diminished responsiveness. Moreover, we performed mast cell degranulation assays in RBL-MRGPRX2 cells that were preincubated with C48/80, CSP-1, or PAMP-12 for 16 h. Consistent with the calcium mobilization results, cells preincubated with CSP-1 and PAMP-12 remained functional, as indicated by substantial β-hexosaminidase release in response to all agonists tested, whereas those preincubated with C48/80 exhibited strongly reduced degranulation responses (**Figure 8H**). To verify that the decreased responsiveness of C48/80-treated cells was not due to cell death, we showed that 16 h preincubation with C48/80 did not affect cell viability (**Figure 8I**). Taken together, these findings demonstrate that prolonged stimulation with different MRGPRX2 agonists differentially influences receptor surface availability and subsequent MC responses.

### Protein synthesis contributes to increased MRGPRX2 expression following prolonged agonist exposure

**Figure 9:**
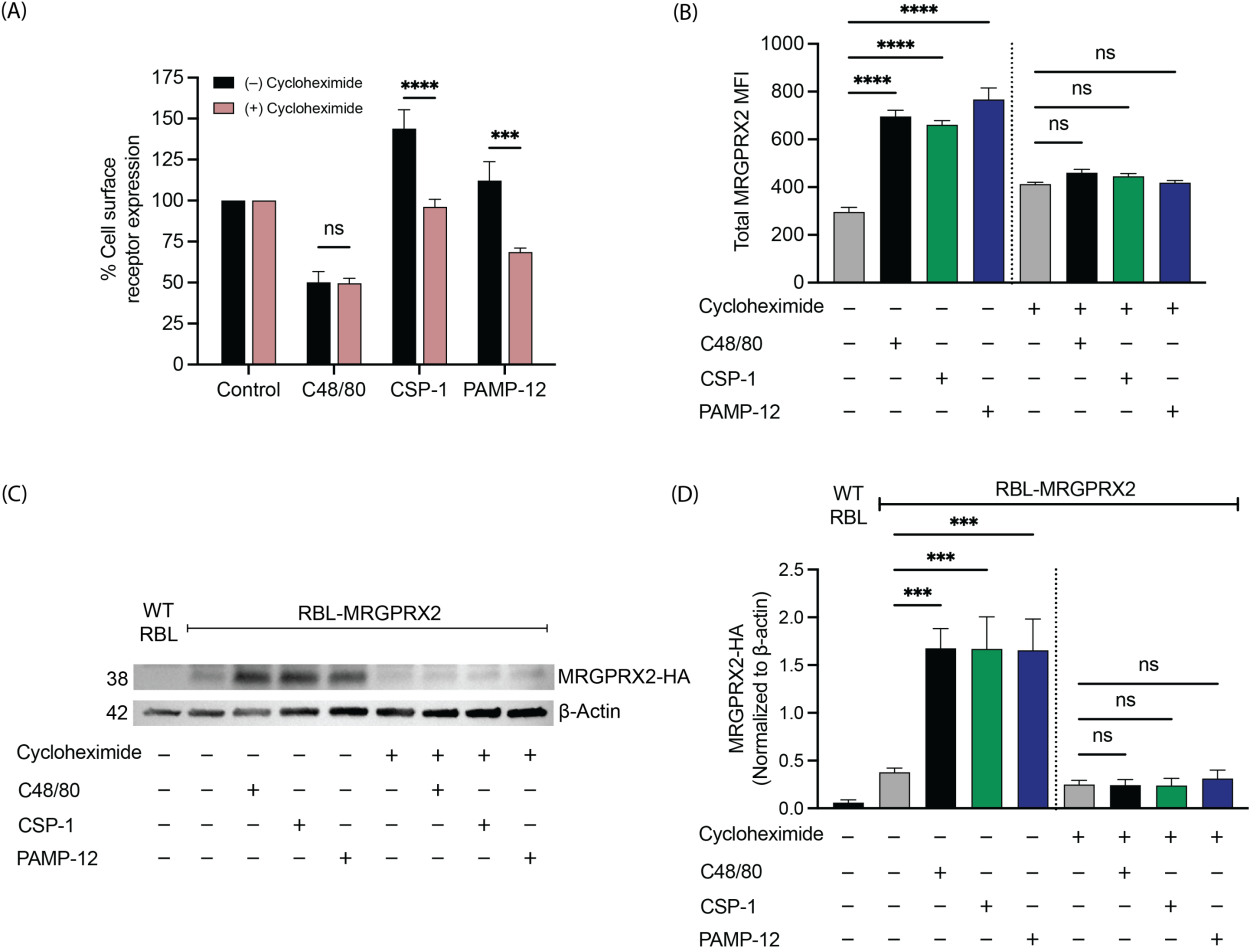
Protein synthesis contributes to increased MRGPRX2 expression following prolonged agonist exposure. RBL-MRGPRX2 cells were incubated with C48/80, CSP-1, or PAMP-12 for 16 h in the absence or presence of cycloheximide (5 µg/mL). **(A)** The cell surface MRGPRX2 expression was determined by flow cytometry and quantitated using a mean fluorescent intensity (MFI) in comparison to the vehicle-treated controls. **(B)** The treated cells were permeabilized, and total MRGPRX2 expression was determined by flow cytometry using mean fluorescence intensity values. **(C)** The treated cells were lysed, cell lysates were collected, and western blotting was performed with anti-HA and anti-β-actin antibodies. Representative blots were shown. **(D)** A bar graph represents the relative intensities of MRGPRX2-HA normalized to the β-actin. Data presented are the mean ± SEM of n = 5–6 independent experiments. Statistical significance was determined by One-way ANOVA and Tukey’s multiple comparisons (for **B, D**) or Two-way ANOVA and Šídák’s multiple comparisons (for **A**) at a value *** p < 0.001, **** p < 0.0001, and ns denotes “not significant”.

To determine whether the increase in cell surface MRGPRX2 expression in CSP-1- and PAMP-12-treated cells involves newly synthesized receptor protein, we utilized cycloheximide, a pharmacological inhibitor of protein synthesis. RBL-MRGPRX2 cells were exposed to CSP-1 or PAMP-12 for 16 h in the presence or absence of cycloheximide co-treatment (5 µg/mL), and cell surface receptor expression was assessed by flow cytometry. We found that the increases in cell surface MRGPRX2 expression induced by CSP-1 and PAMP-12 were significantly reduced by cycloheximide co-treatment (**Figure 9A**). These findings indicate that protein synthesis contributes to the elevated surface expression of MRGPRX2 under these conditions.

It is noteworthy that while cells treated with CSP-1 and PAMP-12 exhibited increased cell surface receptor expression, cells treated with C48/80 did not show a similar increase (**Figure 9A**). To further examine whether prolonged agonist exposure affects overall receptor abundance, we performed a similar experiment in which RBL-MRGPRX2 cells were incubated with C48/80, CSP-1, or PAMP-12 for 16 h with or without cycloheximide co-treatment (5 µg/mL). Cells were then permeabilized using a saponin-based buffer to assess total MRGPRX2 expression by flow cytometry. As shown in **Figure 9B**, treatment with each agonist was associated with increased total MRGPRX2 staining, and this increase was markedly inhibited by cycloheximide. These results suggest that prolonged stimulation with these agonists can promote an overall increase in receptor abundance in a protein synthesis-dependent manner. To further support these observations, RBL-MRGPRX2 cells were exposed to C48/80, CSP-1, or PAMP-12 for 16 h with or without cycloheximide co-treatment (5 µg/mL), and receptor expression was evaluated by Western blot analysis. The MRGPRX2 construct expressed in these cells contains an HA epitope tag, allowing detection with an anti-HA antibody. Consistent with the flow cytometry findings, agonist-treated samples showed increased MRGPRX2-HA signal, whereas cycloheximide co-treatment reduced this effect (**Figures 9C, D**). Collectively, these data support the conclusion that increased MRGPRX2 expression following prolonged agonist exposure is influenced by ongoing protein synthesis.

## Discussion

For the past decade, there has been extensive interest in the studies of the novel MC receptor, MRGPRX2. Growing evidence implicates this receptor in health and diseases ranging from host defense against bacterial infection to allergic contact dermatitis and non-histaminergic itch (7, 8, 44). However, the fact that this receptor is associated with both physiological and pathological conditions suggests that different MRGPRX2 agonists might activate the downstream signaling differently. Che et al. (45), utilized site-directed mutagenesis to generate several MRGPRX2 mutation variants and showed that the neuropeptide SP and PAMP-12 bind to the receptor at different sites and speculated that distinct biological outcomes induced by different agonists might partly depend on the agonists’ binding sites. Recently, the structure of MRGPRX2-agonist complex has been resolved using cryogenic electron microscopy (32, 33). MRGPRX2 has two distinct binding pockets, one shallow pocket that is highly negatively charged and the other pocket that is hydrophobic. Interestingly, while the synthetic peptide ZINC-3573 only binds to MRGPRX2 at the shallow pocket, the neuropeptide cortistatin binds to both pockets (32). These findings suggest that binding and activation of MRGPRX2 by different agonists may be based on the structural component of the peptide agonists, the degree of hydrophobicity, the net charge, as well as how they are folded. It is possible that CSP-1 and PAMP-12 bind to the receptor at different binding pockets and, therefore, contribute to distinct biological outcomes.

Following ligand binding, studies have demonstrated that MRGPRX2-mediated MC activation requires the recruitment of G proteins, especially Gαi and Gαq (28, 35). In this study, we showed that both CSP-1 and PAMP-12 induced MC degranulation mainly via the Gαi and Gαq families of the G proteins. We utilized the BRET2 biosensors and showed that both CSP-1 and PAMP-12 coupled to the same Gαq, Gαi1, and Gαi3 subunits, but not the Gαi2 subunit. This suggests that the differences in CSP-1- and PAMP-12-mediated MRGPRX2 responses do not arise from differences in G-protein coupling specificity.

The extracellular signal-regulated kinases 1 and 2 (ERK1/2) play an important role in a variety of cellular responses, including in MCs. Previous studies have demonstrated that ERK1/2 phosphorylation in MCs can be downstream of GPCR activation, with several lines of evidence supporting a predominant role for G protein-mediated signaling (35, 46). For example, our group has previously reported that ERK1/2 phosphorylation in human MCs is completely abrogated in pertussis toxin-treated cells, suggesting that the G protein signaling is crucial for the activation of ERK1/2 phosphorylation (46). Wang et al. (35) utilized the G protein inhibitors and showed that ERK1/2 phosphorylation in human skin MCs is dependent on the G protein activation. In line with this interpretation, our results show that activation of MRGPRX2 induces robust ERK1/2 phosphorylation that is mediated predominantly through Gαi signaling, irrespective of the agonist used. Together, these findings support a model in which ERK1/2 activation in MCs is largely driven by G protein–dependent mechanisms.

In addition to the G protein pathway, β-arrestin signaling has also been shown to participate in attenuating receptor responsiveness, as well as in the production of chemokines and cytokines (23, 37, 47). Using a transcriptional activation of the luciferase reporter gene to determine the β-arrestin recruitment, we discovered that PAMP-12 strongly induced β-arrestin recruitment to the receptor, whereas CSP-1 did not. This finding is in accordance with the previous report by Chen et al. (31), where they demonstrated greater β-arrestin recruitment in PAMP-12-treated Chinese hamster ovary (CHO) cells transfected with MRGPRX2 than in CSP-1-treated cells using a different β-arrestin recruitment assay. These findings suggest an interesting possibility that the ability of MRGPRX2 agonists to recruit β-arrestin to the activated receptor may play a crucial role in determining MRGPRX2-mediated biological outcomes.

While it is well established that GPCR phosphorylation typically precedes β-arrestin binding and activation, to date, there have been no studies demonstrating MRGPRX2 phosphorylation (40, 41). Our findings provide new insight into the phosphorylation profile of MRGPRX2 and suggest that receptor phosphorylation is both agonist-dependent and spatially restricted. Although a previous study predicted multiple potential serine/threonine phosphorylation sites within the carboxyl-terminus of MRGPRX2, the precise phosphorylation landscape of this receptor has not been previously defined (28). Here, we demonstrate that CSP-1 induces minimal phosphorylation at Ser327, Ser328, and Ser313, whereas PAMP-12 elicits robust phosphorylation at these residues. This differential pattern indicates that distinct agonists may differentially engage receptor kinases or alter receptor conformations in a manner that selectively exposes specific intracellular residues for phosphorylation. Importantly, the weak phosphorylation observed following CSP-1 stimulation suggests that substantial receptor phosphorylation is not required for downstream ERK1/2 activation, which in our system is mediated predominantly through Gαi signaling. Together, these data support a model in which MRGPRX2 signaling outcomes are shaped by agonist-specific phosphorylation patterns that may regulate receptor regulation and signaling bias, rather than serving as a universal prerequisite for effector activation.

**Figure 10:**
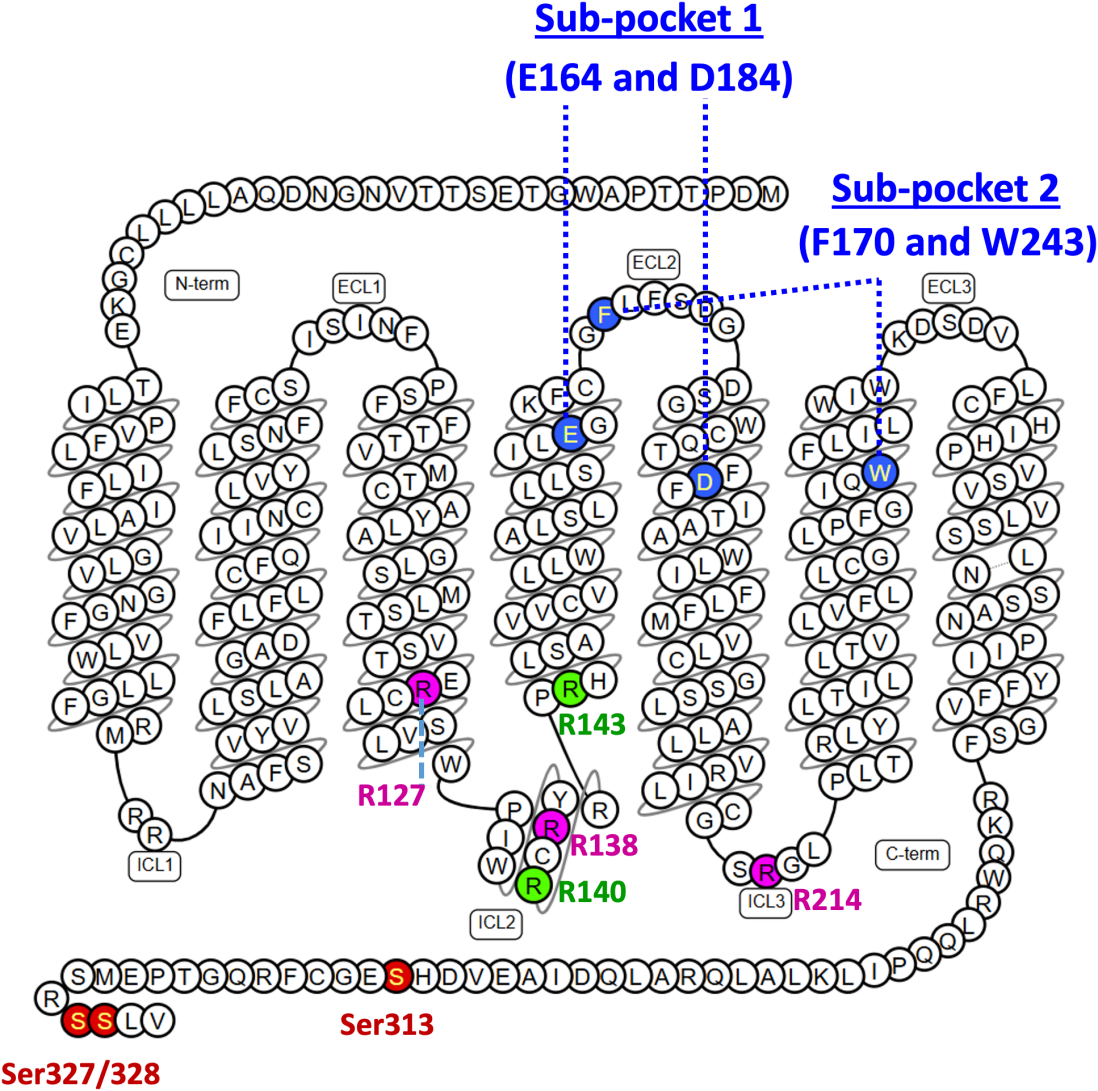
Snake diagram illustrating the secondary structure of MRGPRX2. Each circle represents an individual amino acid residue, labeled using the one-letter amino acid code. Colored backgrounds denote residues of interest: blue circles indicate residues involved in ligand binding; purple circles denote Gαi-coupling residues; green circles indicate Gαq-coupling residues; and red circles represent putative phosphorylation sites.

Cryo-electron microscopy (cryo-EM) studies of MRGPRX2 have revealed that distinct positively charged residues at the cytoplasmic interface selectively mediate G-protein coupling, with Arg127, Arg138, and Arg214 required for Gαi engagement, and Arg140 and Arg143 essential for Gαq coupling (**Figure 10**) (33). While both CSP-1 and PAMP-12 bind MRGPRX2 and induce receptor conformations that support coupling to both Gαi and Gαq, only PAMP-12 promotes receptor phosphorylation and β-arrestin recruitment. Whether these differences arise from unique ligand-receptor interaction modes or reflect distinct conformational states associated with biased signaling remains to be determined.

For most GPCRs, agonist-induced receptor phosphorylation by G protein receptor kinases (GRKs) and the subsequent recruitment of the adapter proteins β-arrestin leads to receptor internalization (10, 48). Whether or not MRGPRX2 undergoes internalization depends on the nature of the ligand used. Recent studies have demonstrated that C48/80, codeine, and substance P cause β-arrestin recruitment and receptor internalization (23, 42). By contrast, the angiogenic HDP AG-30/5C and the HDP mimetic murepavadin weakly activate the β-arrestin signaling and do not cause receptor internalization (20, 21). In this study, we also showed that the degree of β-arrestin recruited to the receptor correlates with the extent of receptor internalization as PAMP-12-treated cells showed ~50-60% reduction in cell surface receptor expression, while those treated with CSP-1 only exhibited ~15-20% reduction. Interestingly, the mechanism via which the cell surface MRGPRX2 undergoes internalization in response to CSP-1 and PAMP-12 has yet to be determined. Lazki-Hagenbach et al. (43) recently showed that the internalization of MRGPRX2 induced by SP is mediated not only via endocytosis but also macropinocytosis. This was discovered through the use of pharmacological inhibitors of clathrin-dependent endocytosis, Arf6-dependent endocytosis, actin polymerization, and a Na^+^/H^+^ exchange inhibitor. Using a potent inhibitor of dynamin, a large GTPase that mediates clathrin-dependent and caveolin-dependent endocytosis, we found that the reduction of cell surface receptors induced by CSP-1 and PAMP-12 is almost completely inhibited, suggesting that dynamin-dependent endocytosis is likely a key mechanism via which MRGPRX2 internalizes in response to both CSP-1 and PAMP-12. However, the involvement of macropinocytosis was not examined in this study.

Desensitization of GPCRs is a complex process that entails the cooperation of multiple regulatory proteins. This process includes an uncoupling of G proteins from the receptor (short-term desensitization) and the downregulation of the receptor or internalization (long-term desensitization) (49). We have previously reported that balanced agonists for MRGPRX2 can desensitize the receptor and attenuate cells’ responsiveness to the same agonist (22, 23). In this study, we identified PAMP-12 as a balanced agonist, whereas CSP-1 is a G protein-biased agonist for MRGPRX2. Similar to what we have previously shown, exposure of the RBL-MRGPRX2 cells to PAMP-12, but not CSP-1, desensitizes the receptor to weaken the response to subsequent stimulation by MRGPRX2 agonists. Interestingly, however, this desensitization effect induced by PAMP-12 appeared to be transient. Prolonged exposure of cells to PAMP-12 was associated with increased cell surface MRGPRX2 expression, which was accompanied by preserved responsiveness upon subsequent stimulation. Therefore, while PAMP-12 can induce short-term receptor desensitization, longer exposure may promote receptor re-expression or altered trafficking that restores functional responsiveness. These findings suggest that MRGPRX2 regulation by balanced agonists may involve dynamic temporal changes rather than sustained desensitization alone. We speculate that the transient receptor desensitization by PAMP-12 likely helps control the inflammation by limiting excessive MRGPRX2 activation, whereas the lack of receptor desensitization by CSP-1 likely helps maintain sufficient MRGPRX2 activation to provide antibacterial host defense function. This finding is interesting because both cases are beneficial to the host since PAMP-12 is associated with inflammatory conditions, and CSP-1 is associated with host defense. Previous studies with MRGPRX2 agonists have implicated the association of the neuropeptide SP with atopic dermatitis and neurogenic inflammation, codeine with clinical pruritus and an experimental inducer of itch, and PAMP-12 with allergic contact dermatitis and non-histaminergic itch (8, 22, 23). On the contrary, murepavadin and CSP-1 have been shown to participate in the host defense (7, 21). Interestingly, it is noteworthy that MRGPRX2 agonists that have been implicated in inflammatory conditions are all balanced agonists, whereas those involved in host defense are G protein-biased agonists. This raises an interesting possibility that β-arrestin activation by balanced MRGPRX2 agonists represents a central difference in MRGPRX2-mediated biological functions. MRGPRX2 agonists that do not recruit β-arrestin or desensitize the receptor may be involved in host protection, whereas those that recruit β-arrestin and desensitize the receptor are likely associated with inflammatory conditions.

Post-endocytic receptor trafficking plays a critical role in determining the fate of internalized GPCRs. For canonical GPCRs, internalized receptors may be sorted into recycling pathways that facilitate return to the plasma membrane, or directed toward lysosomal compartments where degradation can occur (49, 50). A recent study reported that MRGPRX2 internalized in response to substance P remained in a non-degradative intracellular compartment for up to 3.5 h following stimulation (43). In contrast, studies of the β2-adrenergic receptor (β2AR), one of the most extensively characterized class A GPCRs, have shown that receptor trafficking processes such as recycling or degradative sorting can extend over several hours, with outcomes becoming apparent up to 6 h after activation (49, 51). In our study, we observed that MRGPRX2 internalization persisted for as long as 6 h following agonist exposure. Notably, after 16 h of continuous stimulation, cells treated with CSP-1 and PAMP-12 exhibited increased cell surface MRGPRX2 expression, whereas cells treated with C48/80 did not. These findings indicate that prolonged exposure to different agonists leads to distinct patterns of receptor surface availability over time. Interestingly, these differences were also observed among the balanced agonists, as C48/80 and PAMP-12 did not produce identical long-term trafficking outcomes. This suggests that β-arrestin recruitment alone may not fully account for the fate of internalized MRGPRX2 under these conditions. Nevertheless, studies of classical GPCRs indicate that post-endocytic trafficking can be influenced by the stability of the receptor-β-arrestin interaction (52). Dissociation of β-arrestin is often required for receptor dephosphorylation and for progression through recycling pathways, whereas more sustained β-arrestin association has been linked to prolonged intracellular retention. Based on these findings, it is possible that different MRGPRX2 agonists may differentially influence receptor-β-arrestin stability and thereby contribute to the distinct trafficking patterns observed.

One interesting finding from this study is that prolonged stimulation with all tested agonists was associated with an overall increase in total MRGPRX2 expression in a protein synthesis-dependent manner. However, increased surface receptor expression was most evident in CSP-1- and PAMP-12-treated cells, whereas C48/80-treated cells did not show a comparable increase in surface availability. One potential explanation for this difference may relate to the stability and persistence of the agonists themselves. C48/80 is a synthetic polymer, whereas CSP-1 and PAMP-12 are naturally occurring peptides produced by Gram-positive bacteria and keratinocytes, respectively (7, 8). Host defense peptides are generally considered relatively unstable and may exhibit shorter half-lives in biological environments (53, 54). Thus, it is possible that the continued presence of C48/80 could promote sustained receptor activation and internalization, potentially limiting net surface accumulation over prolonged exposure. In contrast, the shorter-lived nature of CSP-1 and PAMP-12 may reduce prolonged receptor engagement, thereby allowing greater surface receptor availability at later time points. Finally, although cycloheximide treatment reduced agonist-associated increases in total MRGPRX2 expression, these experiments do not directly address whether internalized receptors are ultimately routed to degradative pathways. Additional studies examining lysosomal targeting and receptor turnover will be necessary to determine the extent to which degradation contributes to agonist-specific differences in MRGPRX2 trafficking.

Interestingly, when similar internalization and desensitization studies were performed in the LAD2 human mast cell line that endogenously expresses MRGPRX2, increased cell surface MRGPRX2 expression or evidence of new receptor synthesis following prolonged exposure to CSP-1 and PAMP-12 was not observed, and these cells exhibited reduced responsiveness to subsequent stimulation (**Supplementary Figures 1A–C**). These findings suggest that agonist-dependent regulation of MRGPRX2 surface expression may be influenced by cell-specific factors. Differences in proliferation kinetics may partly contribute to this observation, as RBL-2H3 cells proliferate rapidly (doubling approximately every 18 h), whereas LAD2 cells divide much more slowly, with reported doubling times on the order of weeks (55, 56). Such differences in cellular turnover, biosynthetic capacity, or receptor trafficking machinery could influence the extent to which new receptor synthesis restores surface expression after prolonged stimulation. Importantly, although LAD2 cells showed different overall receptor expression dynamics, the trend that CSP-1 treatment was associated with less internalization and desensitization than PAMP-12 remained evident, suggesting that the β-arrestin expression or regulation may differ across cell types. Additional studies in primary human mast cells will be necessary to determine how these mechanisms operate under physiological conditions.

In summary, we demonstrated that CSP-1 and PAMP-12 are MRGPRX2 agonists that similarly activate G protein-mediated signaling through Gαq, Gαi1, and Gαi3, but not Gαi2. Both agonists also comparably induce ERK1/2 phosphorylation and mast cell degranulation. Notably, however, PAMP-12 substantially recruited β-arrestin to the activated receptor, whereas CSP-1 did not. Consistent with this difference, these MRGPRX2 agonists induced receptor internalization to the extent that correlates with the β-arrestin recruitment. Moreover, we demonstrated that PAMP-12 serves as a balanced agonist for MRGPRX2 that desensitizes and cross-desensitizes the receptor to attenuate the response, whereas CSP-1 behaves as a G protein-biased agonist that does not. Interestingly, despite this difference in signaling bias, CSP-1 and PAMP-12 appear to share a similar mechanism for receptor internalization. In addition, prolonged agonist exposure revealed distinct patterns of receptor surface availability and functional responsiveness over time, with protein synthesis contributing to changes in overall receptor expression. Collectively, these findings highlight the presence of biased agonism for MRGPRX2 and suggest that this property may have important clinical implications, including the development of diagnostic or screening strategies for novel MRGPRX2 agonists and the rational design of therapeutic approaches targeting MRGPRX2-mediated conditions.

## Data Availability

The original contributions presented in the study are included in the article/**Supplementary Material**. Further inquiries can be directed to the corresponding author.

## Supplementary material

Supplementary Figure 1 LAD2 cells were exposed to indicated concentrations of C48/80, CSP-1, and PAMP-12 for 16 h. **(A)** The cell surface receptor expression was determined by flow cytometry and quantitated using a mean fluorescent intensity (MFI) in comparison to the vehicle-treated control. **(B)** The treated cells were permeabilized, and total MRGPRX2 expression was determined by flow cytometry using mean fluorescence intensity values. **(C)** The treated cells, for a second time, were exposed to C48/80 (3 µg/mL), CSP-1 (10 µM), and PAMP-12 (10 µM) for an additional 30 min, and β-hexosaminidase release was determined. Data presented are the mean ± SEM of n = 3–6 independent experiments. Statistical significance was determined by One-way ANOVA and Dunnett’s multiple comparisons (for **A**), One-way ANOVA and Tukey’s multiple comparisons (for **B**), or Two-way ANOVA and Dunnett’s multiple comparisons (for **C**) at a value **** p < 0.0001 and ns denotes “not significant”.

## References

[1] Galli SJ Gaudenzio N Tsai M . Mast cells in inflammation and disease: recent progress and ongoing concerns. Annu Rev Immunol. (2020) 38:49–77. doi: 10.1146/annurev-immunol-071719-094903, PMID: 32340580

[2] Fujisawa D Kashiwakura J Kita H Kikukawa Y Fujitani Y Sasaki-Sakamoto T . Expression of Mas-related gene X2 on mast cells is upregulated in the skin of patients with severe chronic urticaria. J Allergy Clin Immunol. (2014) 134:622–33.e9. doi: 10.1016/j.jaci.2014.05.004, PMID: 24954276

[3] Tatemoto K Nozaki Y Tsuda R Konno S Tomura K Furuno M . Immunoglobulin E-independent activation of mast cell is mediated by Mrg receptors. Biochem Biophys Res Commun. (2006) 349:1322–8. doi: 10.1016/j.bbrc.2006.08.177, PMID: 16979137

[4] Plum T Wang X Rettel M Krijgsveld J Feyerabend TB Rodewald HR . Human mast cell proteome reveals unique lineage, putative functions, and structural basis for cell ablation. Immunity. (2020) 52:404–16 e5. doi: 10.1016/j.immuni.2020.01.012, PMID: 32049054

[5] Motakis E Guhl S Ishizu Y Itoh M Kawaji H de Hoon M . Redefinition of the human mast cell transcriptome by deep-CAGE sequencing. Blood. (2014) 123:e58–67. doi: 10.1182/blood-2013-02-483792, PMID: 24671954 PMC3999759

[6] McNeil BD Pundir P Meeker S Han L Undem BJ Kulka M . Identification of a mast-cell-specific receptor crucial for pseudo-allergic drug reactions. Nature. (2015) 519:237–41. doi: 10.1038/nature14022, PMID: 25517090 PMC4359082

[7] Pundir P Liu R Vasavda C Serhan N Limjunyawong N Yee R . A connective tissue mast-cell-specific receptor detects bacterial quorum-sensing molecules and mediates antibacterial immunity. Cell Host Microbe. (2019) 26:114–22.e8. doi: 10.1016/j.chom.2019.06.003, PMID: 31278040 PMC6649664

[8] Meixiong J Anderson M Limjunyawong N Sabbagh MF Hu E Mack MR . Activation of mast-cell-expressed mas-related G-protein-coupled receptors drives non-histaminergic itch. Immunity. (2019) 50:1163–71.e5. doi: 10.1016/j.immuni.2019.03.013, PMID: 31027996 PMC6531358

[9] Serhan N Basso L Sibilano R Petitfils C Meixiong J Bonnart C . House dust mites activate nociceptor-mast cell clusters to drive type 2 skin inflammation. Nat Immunol. (2019) 20:1435–43. doi: 10.1038/s41590-019-0493-z, PMID: 31591569 PMC6858877

[10] Gurevich VV Gurevich EV . GPCR signaling regulation: the role of GRKs and arrestins. Front Pharmacol. (2019) 10:125. doi: 10.3389/fphar.2019.00125, PMID: 30837883 PMC6389790

[11] Cahill TJ 3rd Thomsen AR Tarrasch JT Plouffe B Nguyen AH Yang F . Distinct conformations of GPCR-β-arrestin complexes mediate desensitization, signaling, and endocytosis. Proc Natl Acad Sci U S A. (2017) 114:2562–7. doi: 10.1073/pnas.1701529114, PMID: 28223524 PMC5347553

[12] Shenoy SK Lefkowitz RJ . β-Arrestin-mediated receptor trafficking and signal transduction. Trends Pharmacol Sci. (2011) 32:521–33. doi: 10.1016/j.tips.2011.05.002, PMID: 21680031 PMC3159699

[13] Violin JD Crombie AL Soergel DG Lark MW . Biased ligands at G-protein-coupled receptors: promise and progress. Trends Pharmacol Sci. (2014) 35:308–16. doi: 10.1016/j.tips.2014.04.007, PMID: 24878326

[14] Seyedabadi M Ghahremani MH Albert PR . Biased signaling of G protein coupled receptors (GPCRs): Molecular determinants of GPCR/transducer selectivity and therapeutic potential. Pharmacol Ther. (2019) 200:148–78. doi: 10.1016/j.pharmthera.2019.05.006, PMID: 31075355

[15] Lamichhane R Liu JJ White KL Katritch V Stevens RC Wüthrich K . Biased signaling of the G-protein-coupled receptor β2AR is governed by conformational exchange kinetics. Structure. (2020) 28:371–7.e3. doi: 10.1016/j.str.2020.01.001, PMID: 31978323 PMC7213800

[16] Kim IM Tilley DG Chen J Salazar NC Whalen EJ Violin JD . Beta-blockers alprenolol and carvedilol stimulate beta-arrestin-mediated EGFR transactivation. Proc Natl Acad Sci U S A. (2008) 105:14555–60. doi: 10.1073/pnas.0804745105, PMID: 18787115 PMC2567217

[17] Kim J Grotegut CA Wisler JW Mao L Rosenberg PB Rockman HA . The β-arrestin-biased β-adrenergic receptor blocker carvedilol enhances skeletal muscle contractility. Proc Natl Acad Sci U S A. (2020) 117:12435–43. doi: 10.1073/pnas.1920310117, PMID: 32414934 PMC7275696

[18] Ippolito M Benovic JL . Biased agonism at β-adrenergic receptors. Cell Signalling. (2021) 80:109905. doi: 10.1016/j.cellsig.2020.109905, PMID: 33385503 PMC7878421

[19] Reinartz MT Kälble S Littmann T Ozawa T Dove S Kaever V . Structure-bias relationships for fenoterol stereoisomers in six molecular and cellular assays at the β2-adrenoceptor. Naunyn-Schmiedeberg’s Arch Pharmacol. (2015) 388:51–65. doi: 10.1007/s00210-014-1054-5, PMID: 25342094

[20] Roy S Ganguly A Haque M Ali H . Angiogenic host defense peptide AG-30/5C and bradykinin B(2) receptor antagonist icatibant are G protein biased agonists for MRGPRX2 in mast cells. J Immunol. (2019) 202:1229–38. doi: 10.4049/jimmunol.1801227, PMID: 30651343 PMC6369923

[21] Amponnawarat A Chompunud Na Ayudhya C Ali H . Murepavadin, a small molecule host defense peptide mimetic, activates mast cells via MRGPRX2 and MrgprB2. Front Immunol. (2021) 12:689410. doi: 10.3389/fimmu.2021.689410, PMID: 34248979 PMC8261236

[22] Babina M Wang Z Roy S Guhl S Franke K Artuc M . MRGPRX2 is the codeine receptor of human skin mast cells: desensitization through β-arrestin and lack of correlation with the FcϵRI pathway. J Invest Dermatol. (2021) 141:1286–96.e4. doi: 10.1016/j.jid.2020.09.017, PMID: 33058860 PMC8041898

[23] Chompunud Na Ayudhya C Amponnawarat A Ali H . Substance P serves as a balanced agonist for MRGPRX2 and a single tyrosine residue is required for β-arrestin recruitment and receptor internalization. Int J Mol Sci. (2021) 22:5318. doi: 10.3390/ijms22105318, PMID: 34070125 PMC8158387

[24] Lansu K Karpiak J Liu J Huang XP McCorvy JD Kroeze WK . In silico design of novel probes for the atypical opioid receptor MRGPRX2. Nat Chem Biol. (2017) 13:529–36. doi: 10.1038/nchembio.2334, PMID: 28288109 PMC5391270

[25] Kirshenbaum AS Akin C Wu Y Rottem M Goff JP Beaven MA . Characterization of novel stem cell factor responsive human mast cell lines LAD 1 and 2 established from a patient with mast cell sarcoma/leukemia; activation following aggregation of FcepsilonRI or FcgammaRI. Leuk Res. (2003) 27:677–82. doi: 10.1016/S0145-2126(02)00343-0, PMID: 12801524

[26] Olsen RHJ DiBerto JF English JG Glaudin AM Krumm BE Slocum ST . TRUPATH, an open-source biosensor platform for interrogating the GPCR transducerome. Nat Chem Biol. (2020) 16:841–9. doi: 10.1038/s41589-020-0535-8, PMID: 32367019 PMC7648517

[27] Ali H Richardson RM Tomhave ED DuBose RA Haribabu B Snyderman R . Regulation of stably transfected platelet activating factor receptor in RBL-2H3 cells. Role of multiple G proteins and receptor phosphorylation. J Biol Chem. (1994) 269:24557–63. doi: 10.1016/S0021-9258(17)31428-X 7929127

[28] Chompunud Na Ayudhya C Roy S Alkanfari I Ganguly A Ali H . Identification of gain and loss of function missense variants in MRGPRX2’s transmembrane and intracellular domains for mast cell activation by substance P. Int J Mol Sci. (2019) 20:5247. doi: 10.3390/ijms20215247, PMID: 31652731 PMC6862462

[29] Furuichi K Rivera J Isersky C . The receptor for immunoglobulin E on rat basophilic leukemia cells: effect of ligand binding on receptor expression. Proc Natl Acad Sci U S A. (1985) 82:1522–5. doi: 10.1073/pnas.82.5.1522, PMID: 3156380 PMC397295

[30] Yamaguchi M Lantz CS Oettgen HC Katona IM Fleming T Miyajima I . IgE enhances mouse mast cell Fc(epsilon)RI expression *in vitro* and *in vivo*: evidence for a novel amplification mechanism in IgE-dependent reactions. J Exp Med. (1997) 185:663–72. doi: 10.1084/jem.185.4.663, PMID: 9034145 PMC2196143

[31] Chen E Chuang LS Giri M Villaverde N Hsu NY Sabic K . Inflamed ulcerative colitis regions associated with MRGPRX2-mediated mast cell degranulation and cell activation modules, defining a new therapeutic target. Gastroenterology. (2021) 160:1709–24. doi: 10.1053/j.gastro.2020.12.076, PMID: 33421512 PMC8494017

[32] Cao C Kang HJ Singh I Chen H Zhang C Ye W . Structure, function and pharmacology of human itch GPCRs. Nature. (2021) 600:170–175. doi: 10.1038/s41586-021-04126-6, PMID: 34789874 PMC9150435

[33] Yang F Guo L Li Y Wang G Wang J Zhang C . Structure, function and pharmacology of human itch receptor complexes. Nature. (2021) 600:164–9. doi: 10.1038/s41586-021-04077-y, PMID: 34789875

[34] Knight KM Ghosh S Campbell SL Lefevre TJ Olsen RHJ Smrcka AV . A universal allosteric mechanism for G protein activation. Mol Cell. (2021) 81:1384–96.e6. doi: 10.1016/j.molcel.2021.02.002, PMID: 33636126 PMC8026646

[35] Wang Z Franke K Bal G Li Z Zuberbier T Babina M . MRGPRX2-mediated degranulation of human skin mast cells requires the operation of G(αi), G(αq), Ca++ Channels, ERK1/2 and PI3K-interconnection between early and late signaling. Cells. (2022) 11:953. doi: 10.3390/cells11060953, PMID: 35326404 PMC8946553

[36] Wang Z Franke K Zuberbier T Babina M . Cytokine Stimulation by MRGPRX2 Occurs with Lower Potency than by FcϵRI Aggregation but with Similar Dependence on the Extracellular Signal-Regulated Kinase 1/2 Module in Human Skin Mast Cells. J Invest Dermatol. (2022) 142:414–24.e8. doi: 10.1016/j.jid.2021.07.153, PMID: 34329659

[37] Wang Z Li Z Bal G Franke K Zuberbier T Babina M . β-arrestin-1 and β-arrestin-2 restrain MRGPRX2-triggered degranulation and ERK1/2 activation in human skin mast cells. Front Allergy. (2022) 3:930233. doi: 10.3389/falgy.2022.930233, PMID: 35910860 PMC9337275

[38] DeWire SM Ahn S Lefkowitz RJ Shenoy SK . β-arrestins and cell signaling. Annu Rev Physiol. (2007) 69:483–510. doi: 10.1146/annurev.physiol.69.022405.154749, PMID: 17305471

[39] Kim K Han Y Duan L Chung KY . Scaffolding of mitogen-activated protein kinase signaling by β-arrestins. Int J Mol Sci. (2022) 23:1000. doi: 10.3390/ijms23021000, PMID: 35055186 PMC8778048

[40] Zhou XE He Y de Waal PW Gao X Kang Y Van Eps N . Identification of phosphorylation codes for arrestin recruitment by G protein-coupled receptors. Cell. (2017) 170:457–69.e13. doi: 10.1016/j.cell.2017.07.002, PMID: 28753425 PMC5567868

[41] Dwivedi-Agnihotri H Chaturvedi M Baidya M Stepniewski TM Pandey S Maharana J . Distinct phosphorylation sites in a prototypical GPCR differently orchestrate β-arrestin interaction, trafficking, and signaling. Sci Adv. (2020) 6:eabb8368. doi: 10.1126/sciadv.abb8368, PMID: 32917711 PMC7486103

[42] Babina M Wang Z Roy S Guhl S Franke K Artuc M . MRGPRX2 Is the Codeine Receptor of Human Skin Mast Cells: Desensitization through beta-Arrestin and Lack of Correlation with the FcepsilonRI Pathway. J Invest Dermatol. (2020) 141:1286–1296.e4. doi: 10.1016/j.jid.2020.09.017, PMID: 33058860 PMC8041898

[43] Lazki-Hagenbach P Kleeblatt E Ali H Sagi-Eisenberg R . Spatiotemporal patterns of substance P-bound MRGPRX2 reveal a novel connection between macropinosome resolution and secretory granule regeneration in mast cells. Front Immunol. (2022) 13:892239. doi: 10.3389/fimmu.2022.892239, PMID: 35837385 PMC9273857

[44] Arifuzzaman M Mobley YR Choi HW Bist P Salinas CA Brown ZD . MRGPR-mediated activation of local mast cells clears cutaneous bacterial infection and protects against reinfection. Sci Adv. (2019) 5:eaav0216. doi: 10.1126/sciadv.aav0216, PMID: 30613778 PMC6314830

[45] Che D Zheng Y Hou Y Du X Jia T Zhao Q . Action of substance P and PAMP(9-20) on different excitation sites of MRGPRX2 induces differences in mast cell activation. Int Immunopharmacol. (2021) 101:108342. doi: 10.1016/j.intimp.2021.108342, PMID: 34753104

[46] Vibhuti A Gupta K Subramanian H Guo Q Ali H . Distinct and shared roles of β-arrestin-1 and β-arrestin-2 on the regulation of C3a receptor signaling in human mast cells. PloS One. (2011) 6:e19585. doi: 10.1371/journal.pone.0019585, PMID: 21589858 PMC3093384

[47] Roy S Alkanfari I Chaki S Ali H . Role of MrgprB2 in rosacea-like inflammation in mice: modulation by beta-arrestin 2. J Invest Dermatol. (2022) 142:2988–2997. doi: 10.1016/j.jid.2022.05.005, PMID: 35644498 PMC9634617

[48] Ahamed J Haribabu B Ali H . Cutting edge: Differential regulation of chemoattractant receptor-induced degranulation and chemokine production by receptor phosphorylation. J Immunol. (2001) 167:3559–63. doi: 10.4049/jimmunol.167.7.3559, PMID: 11564766

[49] Rajagopal S Shenoy SK . GPCR desensitization: Acute and prolonged phases. Cell Signal. (2018) 41:9–16. doi: 10.1016/j.cellsig.2017.01.024, PMID: 28137506 PMC5533627

[50] Drake MT Shenoy SK Lefkowitz RJ . Trafficking of G protein-coupled receptors. Circ Res. (2006) 99:570–82. doi: 10.1161/01.RES.0000242563.47507.ce, PMID: 16973913

[51] Shenoy SK Xiao K Venkataramanan V Snyder PM Freedman NJ Weissman AM . Nedd4 mediates agonist-dependent ubiquitination, lysosomal targeting, and degradation of the beta2-adrenergic receptor. J Biol Chem. (2008) 283:22166–76. doi: 10.1074/jbc.M709668200, PMID: 18544533 PMC2494938

[52] Seachrist JL Ferguson SS . Regulation of G protein-coupled receptor endocytosis and trafficking by Rab GTPases. Life Sci. (2003) 74:225–35. doi: 10.1016/j.lfs.2003.09.009, PMID: 14607250

[53] Drayton M Alford MA Pletzer D Haney EF MaChado Y Luo HD . Enzymatically releasable polyethylene glycol – host defense peptide conjugates with improved activity and biocompatibility. J Controlled Release. (2021) 339:220–31. doi: 10.1016/j.jconrel.2021.09.035, PMID: 34597746

[54] Mansour SC Pena OM Hancock RE . Host defense peptides: front-line immunomodulators. Trends Immunol. (2014) 35:443–50. doi: 10.1016/j.it.2014.07.004, PMID: 25113635

[55] Gray EM Díaz-Vázquez G Veatch SL . Growth conditions and cell cycle phase modulate phase transition temperatures in RBL-2H3 derived plasma membrane vesicles. PLoS One. (2015) 10:e0137741. doi: 10.1371/journal.pone.0137741, PMID: 26368288 PMC4569273

[56] Kirshenbaum AS Yin Y Sundstrom JB Bandara G Metcalfe DD . Description and characterization of a novel human mast cell line for scientific study. Int J Mol Sci. (2019) 20:5520. doi: 10.3390/ijms20225520, PMID: 31698677 PMC6888318

