# Supplementary figures and images for: Regulation of MRGPRX2-mediated mast cell function by competence-stimulating peptide 1 and pro-adrenomedullin peptide
Source: Front Immunol. 2026 Mar 17;17:1781889. doi: 10.3389/fimmu.2026.1781889 (PMC13035742; doi:10.3389/fimmu.2026.1781889)

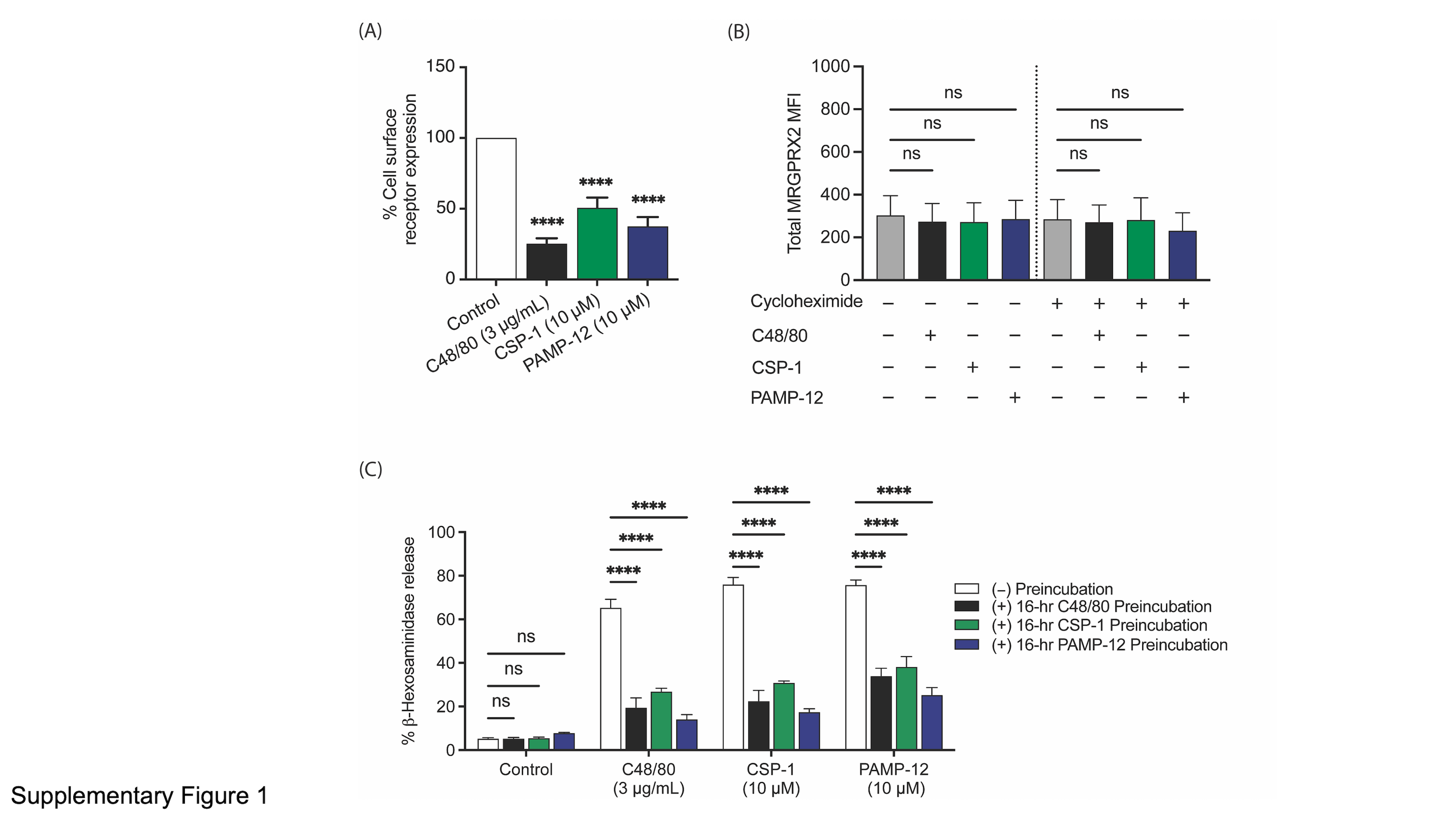

Supplement: Supplementary Figure 1 — LAD2 cells were exposed to indicated concentrations of C48/80, CSP-1, and PAMP-12 for 16 h. (A) The cell surface receptor expression was determined by flow cytometry and quantitated using a mean fluorescent intensity (MFI) in comparison to the vehicle-treated control. (B) The treated cells were permeabilized, and total MRGPRX2 expression was determined by flow cytometry using mean fluorescence intensity values. (C) The treated cells, for a second time, were exposed to C48/80 (3 µg/mL), CSP-1 (10 µM), and PAMP-12 (10 µM) for an additional 30 min, and β-hexosaminidase release was determined. Data presented are the mean ± SEM of n = 3–6 independent experiments. Statistical significance was determined by One-way ANOVA and Dunnett’s multiple comparisons (for A), One-way ANOVA and Tukey’s multiple comparisons (for B), or Two-way ANOVA and Dunnett’s multiple comparisons (for C) at a value **** p < 0.0001 and ns denotes “not significant”. [file Image1.tiff]
